# Structural insight into the human mitochondrial tRNA purine N1-methyltransferase and ribonuclease P complexes

**DOI:** 10.1074/jbc.RA117.001286

**Published:** 2018-06-07

**Authors:** Stephanie Oerum, Martine Roovers, Robert P. Rambo, Jola Kopec, Henry J. Bailey, Fiona Fitzpatrick, Joseph A. Newman, William G. Newman, Albert Amberger, Johannes Zschocke, Louis Droogmans, Udo Oppermann, Wyatt W. Yue

**Affiliations:** From the ‡ Structural Genomics Consortium, Nuffield Department of Clinical Medicine, University of Oxford, Oxford OX3 7BN, United Kingdom,; the § Institut de Recherches Labiris, B-1070 Anderlecht, Belgium,; the ¶ Diamond Light Source, Harwell Science and Innovation Center, Didcot OX11 0QG, United Kingdom,; the ** Manchester Centre for Genomic Medicine, Manchester University NHS Foundation Trust, University of Manchester, Manchester, M13 9WL, United Kingdom,; the ‡‡ Division of Human Genetics, Medical University of Innsbruck, Innsbruck 6020, Austria,; the §§ Laboratoire de Microbiologie, Universite libre de Bruxelles, 1050 Belgium, and; the ¶¶ Botnar Research Centre, NIHR Oxford Biomedical Research Unit, Oxford OX3 7LD, United Kingdom

**Keywords:** ribonuclease P (RNase P), transfer RNA (tRNA), RNA methyltransferase, RNA methylation, complex, HSD10, HSD17B10, MRPP, PRORP, TRMT10C

## Abstract

Mitochondrial tRNAs are transcribed as long polycistronic transcripts of precursor tRNAs and undergo posttranscriptional modifications such as endonucleolytic processing and methylation required for their correct structure and function. Among them, 5′-end processing and purine 9 N1-methylation of mitochondrial tRNA are catalyzed by two proteinaceous complexes with overlapping subunit composition. The Mg^2+^-dependent RNase P complex for 5′-end cleavage comprises the methyltransferase domain–containing protein tRNA methyltransferase 10C, mitochondrial RNase P subunit (TRMT10C/MRPP1), short-chain oxidoreductase hydroxysteroid 17β-dehydrogenase 10 (HSD17B10/MRPP2), and metallonuclease KIAA0391/MRPP3. An MRPP1–MRPP2 subcomplex also catalyzes the formation of 1-methyladenosine/1-methylguanosine at position 9 using *S*-adenosyl-l-methionine as methyl donor. However, a lack of structural information has precluded insights into how these complexes methylate and process mitochondrial tRNA. Here, we used a combination of X-ray crystallography, interaction and activity assays, and small angle X-ray scattering (SAXS) to gain structural insight into the two tRNA modification complexes and their components. The MRPP1 N terminus is involved in tRNA binding and monomer–monomer self-interaction, whereas the C-terminal SPOUT fold contains key residues for *S*-adenosyl-l-methionine binding and N1-methylation. The entirety of MRPP1 interacts with MRPP2 to form the N1-methylation complex, whereas the MRPP1–MRPP2–MRPP3 RNase P complex only assembles in the presence of precursor tRNA. This study proposes low-resolution models of the MRPP1–MRPP2 and MRPP1–MRPP2–MRPP3 complexes that suggest the overall architecture, stoichiometry, and orientation of subunits and tRNA substrates.

## Introduction

The human mitochondrial genome encodes 22 tRNAs that are required for the translation of 13 mitochondrial DNA-encoded proteins (1). Mitochondrial tRNAs ((mt)tRNAs)^5^ are transcribed as long polycistronic transcripts of precursor (mt)tRNAs (pre-(mt)tRNAs) (reviewed in Ref. 2). The individual (mt)tRNAs are released from the polycistronic transcripts by 5′- and 3′-end processing and will undergo post-transcriptional modifications (reviewed in Refs. 3 and 4) that contribute to the correct structure and function (4). Around 100 such post-transcriptional modifications have been identified to 16 nucleobases in mammalian (mt)tRNAs (5, 6), including pseudouridinylation, methylation, and queuosine formation (7).

Methylation of (mt)tRNA occurs on almost all nitrogen sites of the nucleobases and additionally on a few oxygen and carbon atoms (3). The nitrogen-1 (N1) atom of purine bases at position 9 can be methylated to form 1-methyladenosine (m^1^A9) or 1-methylguanosine (m^1^G9) (8). N1-methylation promotes the folding of human (mt)tRNA^Lys^ into the conventional tRNA L-shape (9, 10) and is probably found in 19 of the 22 (mt)tRNAs that contain purine bases at position 9 (8, 11). Across Eukarya and Archaea, formation of both m^1^A9 and m^1^G9 (*i.e.* m^1^R9) is attributed to the Trm10 subfamily of enzymes, belonging to the SPOUT (SpoU and TrmD) superfamily of methyltransferases (MTases) (12, 13). The human genome encodes three Trm10 paralogues with different specificities and subcellular localization: RG9MTD2 (*TRMT10A* gene), RG9MTD3 (*TRMT10B* gene), and RG9MTD1 (*TRMT10C* gene), all representing paralogues to the well-characterized m^1^G9 Trm10 MTases from *Saccharomyces cerevisiae* and *Schizosaccharomyces pombe* (14–16), m^1^A9 Trm10 MTase from *Sulfolobus acidocaldarius*, and m^1^R9 MTase from *Thermococcus kodakaraensis* (17). Of the human paralogues, TRMT10A and TRMT10B are cytosolic m^1^G9 MTases (16), whereas RG9MTD1/TRMT10C is a mitochondrial m^1^R9 MTase (16, 18). The importance of the m^1^R9 modification is underscored by genetic diseases caused by bi-allelic variants in the genes encoding two of the human paralogue enzymes (19–21).

Uniquely among Trm10 members, the m^1^R9 MTase activity of human RG9MTD1/TRMT10C depends on formation of a binary complex with the 17β-hydroxysteroid dehydrogenase type 10 (HSD17B10, also known as SDR5C1) protein (16). HSD17B10 is a well-characterized mitochondrial enzyme of the short-chain dehydrogenase/reductase (SDR) superfamily (22, 23), involved in the third step of isoleucine β-oxidation using NAD^+^ (24, 25). Additionally, HSD17B10 oxidizes a variety of steroids, alcohols, and short straight-/branched-chained fatty acids *in vitro* (26, 27). The role of HSD17B10 in (mt)tRNA methylation is not understood, because neither its NAD^+^/NADH cofactor-binding site nor SDR catalytic residues are directly required for the m^1^R9 MTase activity of TRMT10C (16).

The RG9MTD1/TRMT10C and HSD17B10 proteins are subunits of the human mitochondrial RNase P complex, where they are known as the mitochondrial RNase P protein 1 (MRPP1) and 2 (MRPP2), respectively (28). The active human mitochondrial RNase P requires an additional protein MRPP3, encoded by the *KIAA0391* gene, in complex with MRPP1 and MRPP2. MRPP3 is a Mg^2+^-dependent metallonuclease of the NYN (N4BP1, YacP-like Nuclease) domain family, and is the catalytic component of the RNase P complex that performs the 5′-end cleavage of pre-(mt)tRNAs by phosphodiester bond hydrolysis (28). Human mitochondrial RNase P is a proteinaceous RNase P complex, with no known homology to cytosolic RNase P ribonucleoproteins. Homologues of all three proteins have since been characterized in *Drosophila* (29). MRPP3 homologues functioning as single-protein RNase Ps are called PRORPs (proteinaceous RNase Ps) and are also identified in cytoplasm and organelles from *Arabidopsis thaliana* (30) and mitochondria of *Trypanosoma brucei* (31). Despite sharing component proteins, the methylation and processing of (mt)tRNA by the respective MRPP1–MRPP2 and MRPP1–MRPP2–MRPP3 complexes are not coupled (16).

To date, the molecular basis of MRPP2 involvement in methylation and processing of (mt)tRNA as well as its interaction with partner proteins is not clear, due in part to a lack of structural information beyond that of human MRPP2 (32–34) and Trm10 homologues from yeast and Archaea with low sequence conservation to MRPP1 (15, 35). In this study, we determined the crystal structure of human MRPP1 catalytic domain to understand its methylation mechanism and specificity. To shed light on the two tRNA-modifying activities that MRPP1 and MRPP2 are involved in, we reconstituted the recombinant MRPP1–MRPP2 complex and studied its interaction with tRNA and MRPP3 using biophysical methods.

## Results

### MRPP1 harbors an N-terminal dimerization domain and a C-terminal methyltransferase domain

**Figure 1.**
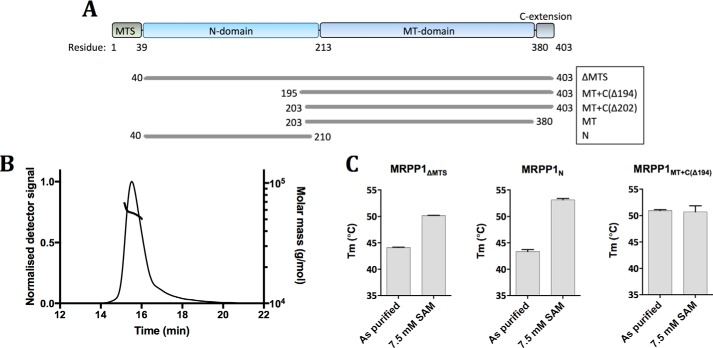
**Biochemical analysis of MRPP1 oligomeric state and SAM binding.** *A*, domain organization of MRPP1: MTS (residues 1–39, *green*), N domain (residues 40–212, *light blue*), MT domain (residues 213–380, *dark blue*), C extension (residues 381–403, *gray*). Also shown are N- and C-terminal boundaries for all constructs in this study. *B*, SEC-MALS curve of MRPP1_ΔMTS_ protein shown as molar mass (g/mol) *versus* elution time (min). *C*, *T_m_* determined by differential scanning fluorimetry, for mature MRPP1 (MRPP1_ΔMTS_) and protein fragments (MRPP1_MT+C(Δ194)_ and MRPP1_N_) in the as-purified form and in the presence of SAM. *, *p* < 0.05; ****, *p* < 0.0001, *n* = 3–4. *Error bars*, S.D.

The 403-amino acid (aa) MRPP1 protein comprises a mitochondrial targeting signal (MTS), an N domain rich in basic residues, a SPOUT-fold methyltransferase (MT) domain, and a ∼20-aa extension at the C terminus (C extension) (Fig. 1*A*). We generated recombinant constructs for MRPP1, including the mature protein (MRPP1_ΔMTS_, aa 40–403), N domain alone (MRPP1_N_, aa 40–210), and MT domain with/without the C extension (MRPP1_MT+C(Δ194)_, aa 195–403; MRPP1_MT+C(Δ202)_, aa 203–403).

The oligomeric states of purified proteins were studied by size-exclusion chromatography (SEC) (Fig. 3*A* and Figs. S3 and S5) using proteins of known molecular weight (*M*_r_) (Fig. S1*A*). MRPP1_ΔMTS_ and MRPP1_N_ eluted at 13.75–13.79 ml (Fig. 3*A*) and 15.79–15.82 ml (Fig. S3*A*), giving rise to apparent masses of 87 and 34 kDa, respectively (calculated monomeric mass of 45,374 Da for MRPP1_ΔMTS_ and 17,825 Da for MRPP1_N_), corresponding to a dimer for both constructs. The elution volume (*V_e_*) of MRPP1_MT+C(Δ194)_ (17.21–17.25 ml; Fig. S3*B*) yields an apparent mass of 18 kDa, consistent with a monomer (calculated monomeric mass 27,109 Da for MRPP1_MT+C(Δ194)_). MRPP1_ΔMTS_ was further assessed using size-exclusion chromatography–coupled multiangle light scattering (SEC-MALS) that determined an apparent mass of 56 ± 1.12 kDa (Fig. 1*B*). These data suggest that isolated MRPP1_ΔMTS_ protein exists as a mixture of monomer and dimer in solution and that dimerization is mediated by the N-terminal region. The yeast and archaeal Trm10 counterparts are reported as monomers in solution even with the presence of N-terminal extensions to their MTase domains (15, 35). Like for other Trm10 proteins, the MTase domain of MRPP1 with the C extension exists as a monomer in solution, consistent with dimerization taking place via the N-terminal region.

MRPP1 contains a SPOUT fold MTase domain known to bind the methylation cofactor *S*-adenosyl-l-methionine (SAM). To confirm that MRPP1 binds SAM, we employed differential scanning fluorimetry (DSF) to determine ligand-induced changes in protein melting temperature (*T_m_*) (Fig. 1*C*). MRPP1_ΔMTS_ and MRPP1_MT+C(Δ194)_ result in dose-dependent *T_m_* increase in the presence of SAM, reaching a maximal *T_m_* shift of 6.04 ± 0.09 and 9.75 ± 0.48 °C, respectively, at 7.5 mm SAM. The *T_m_* of MRPP1_N_, however, was unaffected in the presence of SAM. This confirms that MRPP1 binds SAM within the expected SPOUT MTase fold, and not the N domain. The MTase domain of MRPP1 can also bind SAM homologues, such as the product of methylation *S*-adenosyl-homocysteine (*T_m_* of 5.00 ± 0.18 °C), and nucleotides, such as 3′-AMP (*T_m_* of 3.65 ± 0.16 °C) (Fig. S1*B*).

### The MRPP1 methyltransferase domain adopts a monomeric SPOUT fold

We next characterized MRPP1 by structural methods to understand its molecular properties. Attempts to crystallize *MRPP1*_ΔMTS_ have not been successful, in part due to its tendency to be proteolyzed during the purification procedures. We crystallized *MRPP1*_MT+C(Δ202)_ (Fig. 1*A*) in the presence of SAM, and single-isomorphous replacement with anomalous scattering (SIRAS) was used to determine a 2.49 Å resolution mercury-derivatized structure. A native data set was solved by molecular replacement to 1.96 Å resolution and used for subsequent model building and refinement (Table 1). In the final model, the C-terminal extension (residues 380–403) were disordered and not visible in the electron density. This structure will be referred to hereafter as MRPP1_MT_.

**Table 1:** **Crystallography data processing and refinement statistics for the crystallized MT domain of MRPP1** Values in parentheses are for the highest-resolution shell.

	*HA*	*Native*	*Native*
**Data collection**			
Beamline	Diamond IO4	Diamond IO4	Diamond IO4-I
Space group	P4_3_	P4_3_	P4_3_
Unit cell parameters			
*a*, *b*, *c* (Å)	81.72, 81.72, 146.60	81.44, 81.44, 146.59	82.64, 82.64, 148.62
α, β, γ (degrees)	90, 90, 90	90, 90, 90	90, 90, 90
Molecules/asymmetric unit	3	3	3
Wavelength (Å)	0.97949	0.97949	0.92820
Resolution range (Å)	2.49–53.77 (2.55–2.49)	2.22–81.72 (2.299–2.22)	1.96–58.44 (2.03–1.96)
No. of reflections	911,142 (67,837)	354,295 (24,920)	494,321 (33,562)
No. of unique reflections	33,167 (2450)	51,121 (3773)	71,314 (5279)
Multiplicity	27.5 (27.7)	6.9 (6.6)	6.9 (6.4)
Completeness (%)	98.7 (98.1)	99.97 (99.91)	99.98 (99.97)
*I*/σ(*I*)	12.40 (1.10)	11.75 (1.94)	16.95 (1.48)
CC½	0.998 (0.550)	0.998 (0.659)	0.999 (0.638)
Wilson *B*-factor (Å^2^)	51.89	44.14	42.35
*R*_merge_ (%)	0.202 (3.671)	0.093 (1.312)	0.052 (1.267)
*R*_meas_ (%)	0.210 (3.942)	0.111 (1.587)	0.061 (1.516)
Anomalous completeness	97.9 (95.3)	99.5 (99.3)	99.4 (98.7)
Anomalous multiplicity	13.4 (13.7)	3.3 (3.2)	3.3 (3.0)
Anomalous correlation	0.520 (−0.024)	0.1 (0.001)	0.046 (0.001)

**Refinement**			
*R*_work_ (%)			0.1946 (0.3300)
*R*_free_ (%)			0.2173 (0.3675)
RMSD_bond_ (Å)			0.008
RMSD_angle_ (degrees)			1.23
Ramachandran favored			98
Ramachandran outliers			0
Clash score			3.63
Average *B*-factor (Å^2^) of			54.20
Macromolecules			54.20
Ligands			71.30
Solvent			50.60

**Figure 2.**
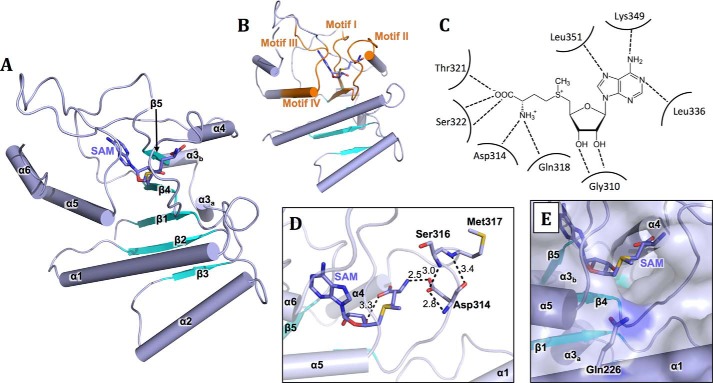
**Crystal structure of MRPP1 methyltransferase domain.** *A*, assignment of secondary structure elements (*blue*, helices α1–6; *cyan*, strands β1–5) in the *cartoon representation* of MRPP1 MT domain (MRPP1_MT_). SAM ligand is shown in *sticks. B*, the four conserved motifs I–IV (*orange*) in the Trm10 family, to which MRPP1 belongs, are mapped on the MRPP1_MT_ structure. SAM is shown in *sticks. C*, schematic representation of residues interacting with SAM. *Dashed lines* represent hydrogen bonds between residues and the ligand. *D*, view of the active site highlighting the role and interactions of the strictly conserved Asp-314 with both the ligand SAM and the backbone of residues Ser-316 and Met-317. Hydrogen bonds are indicated as *dashed lines*, and bonding distances (Å) are shown. *E*, surface representation of the active site highlighting the strictly conserved residue Gln-226, with its side chain pointing toward the SAM ligand.

MRPP1_MT_ displays the canonical SPOUT α/β fold, consisting of a central 6-stranded β-sheet (β1–β6) flanked on both sides by α-helices (α1–α6) (Fig. 2*A*). The SPOUT fold is characterized by the trefoil knot near the C terminus, forming a deep crevice that accommodates the active site. The closest structural homologues of MRPP1 (Fig. S1*C*), based on a DALI search, are from Trm10 family members that include human TRMT10A (PDB entry 4FMW, *Z*-score 26.4, C^α^ RMSD of 1.5 Å, 26% sequence identity), Trm10 from *S. pombe* (4JWF, 25.4, 1.7 Å, 28%), Trm10 from *S. cerevisiae* (4JWJ, 23.6, 1.9 Å, 23%), and Trm10 from *S. acidocaldarius* (5A7T, 14.0, 2.5 Å, 16%).

Although MRPP1_MT_ crystallizes with three molecules in the asymmetric unit (C^α^ RMSD of 0.204–0.238 Å), PISA intermolecular surface analysis (36) of the three molecules did not reveal interactions that could result in stable, physiologically relevant oligomer formation. Most characterized SPOUT domains are dimers, mediated by helix α5 as the dimerization interface. Like other Trm10 members, helix α5 packs against an additional helix (α6) in our MRPP1_MT_ structure and hence is sterically blocked from forming the typical SPOUT dimer interface (Fig. S1*D*). Therefore, our structural observations are consistent with analytical SEC of the isolated MRPP1_MT_ domain, which elutes as a monomer (Fig. S3*B*), and with other Trm10 member proteins characterized to date (Trm10 from yeast and Archaea) (15, 35).

### SAM-bound MRPP1 structure reveals active site and substrate binding surface

Each MRPP1_MT_ subunit is bound by a SAM molecule (Fig. 2A and Fig. S1*E*). SAM adopts an L-shaped bent configuration, in a binding pocket close to the trefoil knotted region of the SPOUT fold. The SAM pocket is enclosed by four extended loops highly conserved in sequence (Fig. S2), harboring motifs I (aa 288–294), II (aa 307–323), III (aa 335–338), and IV (aa 350–360) (Fig. 2*B*). The SAM adenine moiety (N1, N7, and exocyclic N6 atoms) forms main-chain hydrogen bonds to backbone atoms of Leu-336, Lys-349, and Leu-351 (Fig. 2*C*). The methionine moiety carboxyl group forms side-chain hydrogen bonds to Asp-314 and Ser-322 and main-chain hydrogen bonds to backbone atoms of Ser-322, Thr-321, and Gln-318. The ribose 2′-3′-OH moiety forms a hydrogen bond with Gly-310 backbone amide. The methyl Cϵ atom of SAM is accessible through a shaft that opens to an extensive surface of positive charges (Lys-216, Arg-217, Lys-218, Arg-235, Arg-236, Arg-256, Lys-260, Arg-261, Lys-265, Lys-268, Lys-315, Arg-358, Lys-378, and Arg-379), which may assist in substrate tRNA binding.

The crystal structures of m^1^R MTases, including Trm10 (m^1^G9) and TrmD (m^1^G37), feature a conserved aspartate residue suggested to act as the general base to abstract a proton from the N1 atom of guanosine and promote methylation (37). For *S. cerevisiae*, the corresponding aspartate residue was proposed to act together with other carboxylate-containing residues to promote ionization required for catalysis (37). For adenosine, this residue has been suggested to abstract a proton from the exocyclic N6 atom, forming an imino tautomer (38, 39). In MRPP1, residue Asp-314 is strictly invariant among 150 orthologues and hydrogen-bonds to the backbone amide of Ser-316, Met-317, and the NH_3_^+^ group of the SAM methionyl moiety (Fig. 2*D*). The distance of 4 Å between Asp-314 and the transferred methyl carbon (Cϵ) atom of SAM renders it likely to function in proton abstraction. Another active-site residue of MRPP1 strictly invariant across orthologous proteins is Gln-226, located in helix α1 with its side chain pointing toward the active site (Fig. 2*E*).

### MRPP1 uses multiple regions to interact with MRPP2

**Figure 3.**
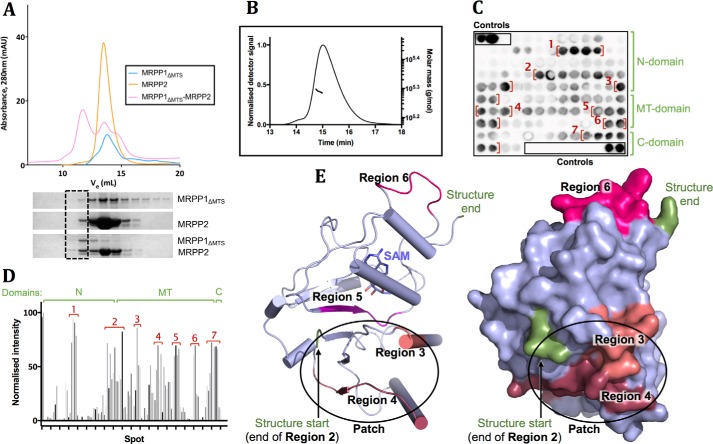
**Interactions between MRPP1_ΔMTS_ and MRPP2.** *A*, analytical SEC profile of MRPP1_ΔMTS_ and MRPP2 applied individually (*blue* and *orange line*, respectively) or in a mixture (*black line*) on a Superdex S200 column. The *black box* indicates lanes where complexed proteins would be found on an SDS-polyacrylamide gel of eluted fractions. A replicate of this experiment is shown in Fig. S5. *B*, SEC-MALS curve of the complex between MRPP1_ΔMTS_ and MRPP2 shown as molar mass (g/mol) *versus* elution time (min). *C*, a SPOT membrane of MRPP1_ΔMTS_ with bound His_6_-tagged MRPP2 immunoblotted with anti-His antibody and visualized by chemiluminescence. The membrane contains an array of 15-mer MRPP1_ΔMTS_ peptides, together covering the full sequence of MRPP1_ΔMTS_. The controls of His_6_ peptide (*black*) or no peptide coupled (*white*) are shown in *black boxes. Red brackets* indicate regions were MRPP2 is identified to interact with MRPP1. MRPP1 domains are indicated in *green*. The *blue dashed line* indicates the crystallized MT-domain (MRPP1_MT_) region. *D*, densitometry analysis of each spot on the membrane shown as a *bar chart* of spot intensity. Regions of interactions and MRPP1 domains (N domain, MT domain, and C extension) are indicated as for *C. E*, mapping of MRPP2 interaction regions onto the crystal structure of MRPP1_MT_ shown in *cartoon* (*left*) and *surface* (*right*) *representation*. Regions 3 (*salmon*) and 4 (*cherry*) form a patch near part of region 2. The start and end residues of the MRPP1_MT_ structure are *labeled* (*green*).

The expression and stability of MRPP1 in vivo depends on MRPP2 (40), implicating a physical complex between MRPP1 and MRPP2 as previously demonstrated (16, 28). When recombinant MRPP1_ΔMTS_ is applied to analytical SEC with excess MRPP2 (Fig. 3*A* and Fig. S5), this results in a peak (*V_e_* = 11.72 ml) of the complex and two subsequent peaks corresponding to uncomplexed single components (*V_e_* = 13.48 ml for MRPP1_ΔMTS_, *V_e_* = 13.79 ml for MRPP2). The complexed proteins retained their ability to bind the cofactor (NADH) and methyl donor (SAM), as shown in a DSF assay (Fig. S1*F*). Further, when MRPP2 is mixed with either MRPP1_N_ (Fig. S3*A*) or MRPP1_MT(Δ194)_ (Fig. S3*B*), no complex peaks were observed. This suggests that both the N and MT domains of MRPP1 are required for interaction with MRPP2.

We next determined the biophysical and enzymatic activities of the MRPP1_ΔMTS_–MRPP2 complex, co-expressed using an *Escherichia coli* bicistronic system, such that MRPP1_ΔMTS_ is His-tagged at the N terminus and MRPP2 is untagged. MRPP2 is co-purified only with His-MRPP1_ΔMTS_ on affinity chromatography resin, and not with His-MRPP1_N_ or His-MRPP1_MT(Δ194)_ (Fig. S3*C*). This agrees with analytical SEC findings that both the N and MT domains of MRPP1 are required for interaction with MRPP2. Co-expression of MRPP1 and MRPP2 also eliminated degradation otherwise observed for MRPP1_ΔMTS_ when expressed alone (Fig. S3*D*). SEC-MALS of the MRPP1_ΔMTS_–MRPP2 complex reveals a mass of 194 ± 1.16 kDa (Fig. 3*B*), indicating a stoichiometry of 2 MRPP1_ΔMTS_ monomers and 4 MRPP2 monomers in the complex with an expected mass of 208 kDa.

To map the sequence region(s) of MRPP1 that interacts with MRPP2, we probed an array of serially overlapping 15-mer peptides of the entire MRPP1 sequence, generated using SPOT technology (41), with His-tagged MRPP2 followed by immunoblotting. Seven footprints indicative of putative binding regions (Fig. 3C) are found across both domains of MRPP1, with the strongest binding peptides covering the stretch of aa 100–105 in the N domain (Fig. 3*D*, region 1). Additional binding peptides are found between the end of the N domain and start of the MT domains (hereafter referred to as the linker) (region 2), within the MT domain (regions 3–6), and in the C extension (region 7).

### When mapped onto the MRPP1_203–380_ structure, regions 3, 4, and 6 are surface-exposed

Regions 3 and 4 are closer to the start of the structure and cluster into a patch with region 2 in the linker (Fig. 3*E*) seven residues away. Region 6 is located in a mobile loop near the end of the structure, and the equivalent region in yeast Trm10s has been shown to play a role in activity (15). In summary, regions from the N domain, MT domain, and C extension of MRPP1 are all involved in interactions with MRPP2, which agrees with the SEC and co-expression data.

### The N domain of MRPP1 is involved in tRNA binding

**Figure 4.**
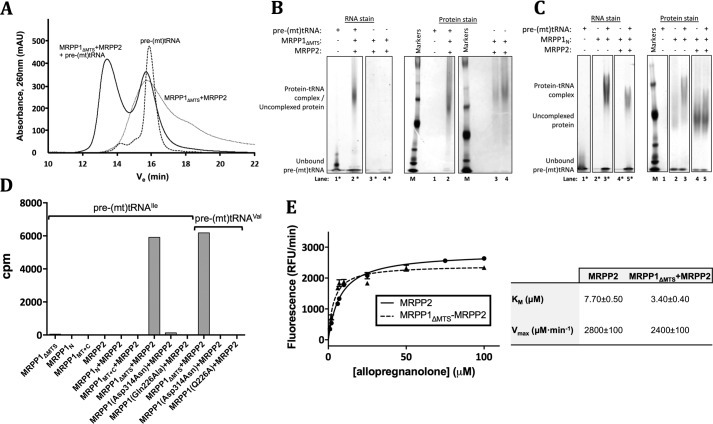
**tRNA binding and activity of MRPP1_ΔMTS_ and MRPP2.** *A*, SEC-HPLC profiles of pre-(mt)tRNA^Ile^ (*black dashed line*), the complex of MRPP1_ΔMTS_ and MRPP2 (gray *dashed line*), and a combination of all three components (*solid black line*) to indicate a shift in elution volume of the complex in the presence of tRNA. *B*, native PAGE nondenaturing gels of pre-(mt)tRNA^Ile^ alone (*lanes 1* and 1*), a mixture of pre-(mt)tRNA^Ile^ and the complex of MRPP1_ΔMTS_ and MRPP2 (*lanes 2* and 2*), and the complex of MRPP1_ΔMTS_ and MRPP2 loaded at the same concentration as in *lanes 2* and 2* (*lanes 4* and 4*) and at half that concentration (*lanes 3* and 3*). The gel was stained for RNA (lanes 1*–4*) and protein (*lanes 1–4*), including *M*_r_ protein markers (*M*). Lanes shown are taken from two gels, with the protein marker lane used for lane alignment. *C*, native PAGE nondenaturing gels stained similarly as for *B*, containing samples of pre-(mt)tRNA^Ile^ alone (*lanes 1* and 1*), MRPP1_N_ alone (*lanes 2* and 2*), a mixture of pre-(mt)tRNA^Ile^ and MRPP1_N_ (*lanes 3* and 3*), a mixture of MRPP1_N_ and MRPP2 (*lanes 4* and 4*), and a mixture of pre-(mt)tRNA^Ile^, MRPP1_N_, and MRPP2 (*lanes 5* and 5*). The gel sections were stained for RNA (lanes 1*–5*) and protein (*lanes 1–5*), including *M*_r_ protein markers (*M*). Lanes shown are taken from one gel, with the protein marker lane used for lane alignment. *D*, measurement of m^1^G MTase (substrate: pre-(mt)tRNA^Ile^) and m^1^A MTase (substrate: pre-(mt)tRNA^Val^) activities for MRPP1_ΔMTS_ and various individual domains and catalytic mutants (D314N and Q226A) applied with and without MRPP2, shown as a *bar chart* of the measured radioactivity (cpm) incorporated into tRNA. *E*, measurement of SDR activity of MRPP2 alone and in complex with MRPP1_ΔMTS_, using the substrate allopregnanolone, shown as a plot of the relative fluorescence (relative fluorescence units (*RFU*)/min) against the concentration of substrate (μm). Kinetic parameters for the *K_m_* and maximal velocity (*V*_max_) were calculated from Michaelis–Menten fitting of the shown curve using GraphPad Prism. *mAU*, milli-absorbance units.

Unlike Trm10s from lower eukaryotes, the m^1^R9 MTase activity of MRPP1 requires the presence of MRPP2 in a binary complex (16). We first confirmed the binding of tRNA to the MRPP1_ΔMTS_–MRPP2 complex in size-exclusion HPLC, resulting in a separate peak for a protein–tRNA complex (Fig. 4*A*). Binding of tRNA to MRPP1–MRPP2 was also confirmed by an electrophoretic mobility shift assay (EMSA) (Fig. 4*B*), as shown by a shift of the RNA-stained band when bound to MRPP1_ΔMTS_–MRPP2. It was previously shown in an EMSA that MRPP1 can bind the tRNA substrate (16). Here, our EMSA also revealed a shift of the RNA-stained band in the presence of MRPP1_N_ alone (Fig. 4*C*), indicating that this domain is involved in tRNA binding.

### MRPP2 is required for MRPP1 MTase function, but MRPP1 does not inhibit MRPP2 activity

We next measured *in vitro* tRNA MTase activity using [*methyl*-^3^H]SAM and purified tRNAs (pre-(mt)tRNA^Ile^ for m^1^G9 MTase activity and pre-(mt)tRNA^Val^ for m^1^A9 MTase activity) as substrates. Co-expressed MRPP1_ΔMTS_–MRPP2 complex, but not MRPP1_ΔMTS_ or MRPP1_MT(Δ202)_ alone, methylates pre-(mt)tRNA^Ile^ and pre-(mt)tRNA^Val^ (Fig. 4*D*). Importantly, both m^1^G9 and m^1^A9 MTase activities of the complex are abolished with the D314N or Q226A substitution on MRPP1 (Fig. 4*D*), confirming the essential catalytic roles of the strictly invariant Asp-314 and Gln-226 residues. Mixtures of MRPP1_MT_ + MRPP2 and MRPP1_N_ + MRPP2 proteins, which did not result in complex formation in SEC (Fig. S3, *B* and *C*), also did not exhibit tRNA MTase activity (Fig. 4*D*).

We also determined whether the presence of MRPP1 would influence the SDR function of MRPP2 within the MRPP1_ΔMTS_–MRPP2 complex. The *in vitro* 3α-HSD activity of the complex, measured using the neurosteroid allopregnanolone as MRPP2 substrate, revealed a *V*_max_ similar to that of MRPP2 protein alone (Fig. 4*E*). Further studies are warranted to determine whether the 2-fold reduced *K_m_* (allopregnanolone) of the complex, compared with MRPP2 alone, would indicate enhancement of MRPP2 activity by MRPP1. In summary, MRPP2 is required for methyltransferase activity of MRPP1, but MRPP2 within the MRPP1–MRPP2 complex is still fully functional as an SDR.

### MRPP1–MRPP2 complex interacts with MRPP3 in the presence of tRNA

**Figure 5.**
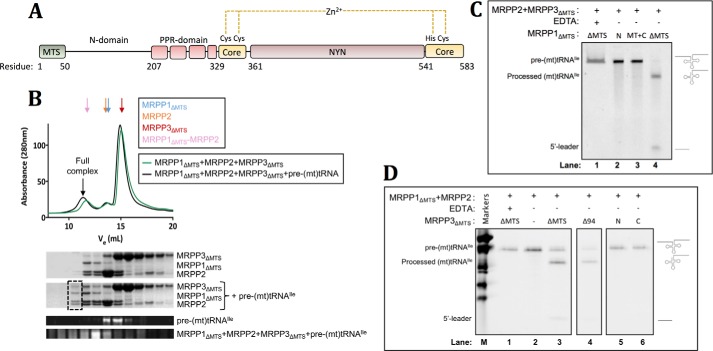
**Characterization of MRPP3_ΔMTS_ alone and in complex with MRPP1_ΔMTS_ and MRPP2.** *A*, domain organization for MRPP3_ΔMTS_ indicating the N extension (residues 50–206), PPR domain (residues 207–329, *red*), core domain (residues 330–361 and 542–583, *yellow*), NYN domain (residues 362–541, *brown*), and structural zinc ion bound in a Cys-Cys-His-Cys motif in the core domain. *B*, *top*, analytical SEC profiles for the mixture of MRPP1_ΔMTS_, MRPP2, and MRPP3_ΔMTS_ (*green line*) and the mixture of MRPP1_ΔMTS_, MRPP2, MRPP3_ΔMTS_, and pre-(mt)tRNA^Ile^ (*black line*) applied to a Superdex S200 column. A replicate of this experiment is shown in Fig. S5. The elution volumes for individual MRPP1_ΔMTS_ (from Fig. 3*A*), MRPP2 (from Fig. 3*A*), and MRPP3_ΔMTS_ (from Fig. S4*F*) proteins, as well as the mixture of MRPP1_ΔMTS_ and MRPP2 (from Fig. 3*A*), are indicated with *arrows above* the chromatogram (*blue*, *orange*, *red*, and *pink*, respectively). *Bottom*, SDS-PAGE (*first two gel panels*) and urea-PAGE (*last two gel panels*) analysis of eluted fractions to visualize proteins and pre-(mt)tRNA^Ile^, respectively. The *black dashed box* indicates lanes where the complex containing MRPP1_ΔMTS_, MRPP2, MRPP3, and pre-(mt)tRNA^Ile^ would be found on the SDS-PAGE. *C*, urea-polyacrylamide denaturing gels showing the RNase P reaction of pre-(mt)tRNA^Ile^ set up, for mixtures of MRPP2 and MRPP3_ΔMTS_ with different MRPP1 truncated proteins (MRPP1_ΔMTS_, MRPP1_MT+C(Δ202)_, MRPP1_N_). All lanes shown are taken from one experimental gel only. A downward shift in band location of processed (mt)tRNA^Ile^ relative to that of pre-(mt)tRNA^Ile^ and the concomitant appearance of a band corresponding to the removed 5′-leader indicate RNase P activity. *D*, urea-polyacrylamide denaturing gels showing the RNase P reaction of pre-(mt)tRNA^Ile^ set up for the complex of MRPP1_ΔMTS_ and MRPP2 without MRPP3 (*lane 2*) and in the presence of different truncated MRPP3 proteins (*lanes 3–6*). A negative control of MRPP1_ΔMTS_, MRPP2, and MRPP3_ΔMTS_ mixed with EDTA (*lane 1*) is included. *M*_r_ RNA markers are shown (*lane M*). All lanes shown are taken from one experimental gel only. Reactions in *C* and *D* were run for 30 min at protein concentrations of 300 nm MRPP1–MRPP2 and 150 nm MRPP3.

The MRPP1–MRPP2 complex interacts with the Mg^2+^-dependent endoribonuclease MRPP3, resulting in the ternary RNase P complex that performs 5′-end pre-(mt)tRNA processing (28). The mature MRPP3 protein (MRPP3_ΔMTS_) contains an N extension (aa 51–206), a pentatricopeptide repeat (PPR) domain (aa 207–328), the first half of a core domain (aa 329–360), a catalytic NYN domain (aa 360–541), and the second half of the core domain (aa 342–583) (Fig. 5*A*). MRPP3 is expected to be the catalytic subunit of this RNase P complex, although it is unclear how MRPP3 interacts with the binary complex of MRPP1–MRPP2 or the pre-(mt)tRNA substrate.

We reconstituted the ternary complex (Fig. 5*B* and Fig. S5) by mixing MRPP3 (Fig. S4*A*) with MRPP1 and MRPP2 that were either expressed individually or co-expressed. A preincubated mixture of MRPP1_ΔMTS_, MRPP2, and MRPP3_ΔMTS_ was applied to an SEC column, which eluted predominantly as peaks corresponding to the MRPP1–MRPP2 complex and individual proteins, and not the ternary complex (Fig. 5*B*, *top*, *green line*). However, a mixture of the three proteins incubated with excess pre-(mt)tRNA gave rise to an earlier elution peak than the binary complex, indicating a larger *M*_r_ species (Fig. 5*B*, *top*, *black line*). SDS-PAGE analysis confirmed the presence of all three proteins in this peak (Fig. 5*B*, *bottom*). Therefore, the interaction between MRPP3_ΔMTS_ and its two partner proteins, MRPP1_ΔMTS_ and MRPP2, is facilitated by the presence of its pre-tRNA substrate. The C-terminal region of MRPP3 (aa 274–583), harboring the endonuclease domain for the RNase P activity, is not sufficient for complex formation with MRPP1, MRPP2, and pre-(mt)tRNA^Ile^ (Fig. S4*B*).

The *in vitro* RNase P activity of the reconstituted MRPP1–MRPP2–MRPP3 complex was measured by probing the formation of 5′-end cleavage products of the pre-(mt)tRNA^Ile^ substrate. The assay was set up with complex samples reconstituted from mature proteins, and fragments of MRPP1 (Fig. 5*C*) and MRPP3 (Fig. 5*D*). Detection of substrate and cleavage products was carried out with a denaturing urea-polyacrylamide gel stained for RNA. The presence of processed (mt)tRNA and 5′-leader by-product was observed in the reaction lane containing all three proteins together with the pre-(mt)tRNA substrate (Fig. 5*D*, *lane 3*), confirming RNase P activity for the reconstituted ternary complex. Consistent with previous studies (28), MRPP3 protein alone did not show any RNase P activity toward pre-(mt)tRNA^Ile^ (Fig. S4*C*). RNase P activity was also observed (*lane 4*) when the MRPP3 component of the ternary complex was replaced by a slightly truncated protein at the N terminus (Δ94, aa 95–583), but not when replaced by either the N-terminal half of MRPP3 (*N*, aa 86–274) that contains the putative tRNA-binding PPR domain (*lane 5*) or the C-terminal half (*C*, 274–583) that contains the endonuclease and core domains (*lane 6*). Nor was RNase P activity observed when MRPP1_ΔMTS_ was replaced by MRPP1_N_ or MRPP1_MT+C(Δ202)_ (Fig. 5*C*). Therefore, the RNase P activity of the complex requires multiple regions of the MRPP1 and MRPP3 intact proteins, and not their isolated domains.

### Small angle X-ray scattering (SAXS) modeling of individual proteins and complexes

**Figure 6.**
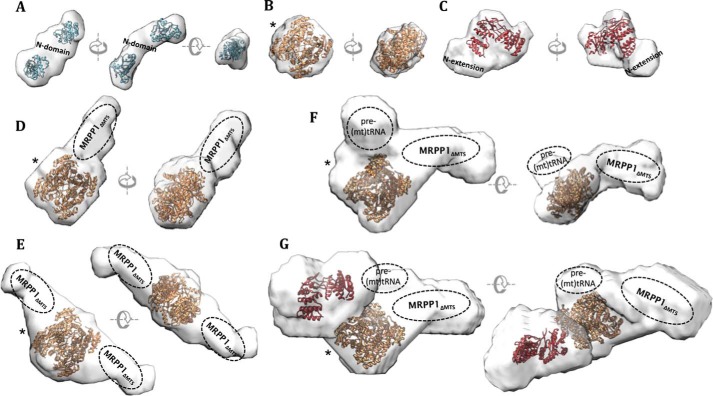
**Low-resolution models from small angle X-ray scattering.** Shown are *ab initio* envelopes derived from scattering data for MRPP1_ΔMTS_ (*A*), MRPP2 (*B*), MRPP3_ΔMTS_ (*C*), complex containing MRPP1_ΔMTS_ and MRPP2 processed in point group *P1* (*D*), complex containing MRPP1_ΔMTS_ and MRPP2 processed in point group *P2* (*E*), MRPP1_ΔMTS_–MRPP2–(pre)tRNA^Ile^ processed in point group *P1* (*F*), MRPP1_ΔMTS_–MRPP2–(pre)tRNA–MRPP3_ΔMTS_ processed in point group *P1* (*G*). In *A*, the three views are related by 90° rotations along the *x* axis and *y* axis. For *B–D*, the two views are related by 90° rotations along the *x* axis, and for *E–G*, the two views are related by 90° along the *y* axis. Crystal structures, wherever available, are fitted into the envelopes for MRPP2 (*orange*; PDB entry 1U7T) and MRPP3_Δ206_ (*red*; PDB entry 4XGL), and MRPP1_MT_ (*blue*; this study) in the situation where this exists as a dimer. In empty spaces of the envelopes where no crystal structures were fitted, the putative locations for certain subunits or domains are proposed. *, views where MRPP2 (*orange*) is shown in the same orientation.

We utilized SAXS to characterize the solution state of the binary complex (MRPP1_ΔMTS_–MRPP2) as well as the binary and ternary complexes in the presence of pre-(mt)tRNA^Ile^ (MRPP1_ΔMTS_–MRPP2–tRNA and MRPP1_ΔMTS_–MRPP2-MRPP3_ΔMTS_–tRNA, respectively). To ensure that scattering data were measured on monodisperse samples, SAXS measurements were performed as analytical, size-exclusion chromatographic separations connected in-line to a SAXS flow cell. The scattering data for the various complexes were collected (Fig. S6, *C*, *D*, and *G*) and processed (Fig. S7) to determine the radius of gyration (*R_g_*), particle volume, and maximal intraparticle dimension (*D*_max_) values (Table 2). Shape restoration from the scattering data yielded *ab initio* molecular envelopes for the three complexes (Fig. 6, *D*, *E*, *F*, and *G*).

**Table 2:** **Parameters derived from small angle x-ray scattering studies of MRPP1_ΔMTS_, *MRPP2, MRPP3*_ΔMTS_, *and their complexes*** *Ab initio* model-building statistics are given. Guinier *R_g_* < 1.3 for all data sets. *I*(0), forward scattering; *R_g_*, radius of gyration; a.u., arbitrary units; *RS-R_g_*, real-space radius of gyration; Porod *V*, Porod volume; *D*_max_, maximal intraparticle dimension. a.u., absorbance unit.

	*MRPP1*	*MRPP2*	*MRPP3*	Δ*206-MRPP3*	*MRPP1-MRPP2*	*MRPP1-MRPP2- pre-tRNA*^Ile^	*MRPP1-MRPP2-MRPP3- pre-tRNA*^Ile^
Guinier *R_g_*	40.10 ± 2.89	29.24 ± 0.95	33.79 ± 1.15	33.30 ± 1.52	46.77 ± 1.16	72.71 ± 5.45	108.8 ± 7.25
*I*(0) (×10^−3^ a.u.)	3.02 ± 0.29	9.07 ± 0.25	9.23 ± 4.68	2.87 ± 0.46	23.42 ± 0.71	21.71 ± 1.53	42.87 ± 4.01
RS-*R_g_* (Å)	34.40 ± 1.88	28.44 ± 0.44	33.18 ± 1.12	28.83 ± 1.47	45.59 ± 0.97	69.67 ± 2.77	82.60 ± 0.95
Porod *V* (Å^3^)	174,930	167,454	306,000	167,576	873,102	1,279,622	4,077,408
*D*_max_ (Å)	100	80	111	98	154	216	248

**Model building**							

Point symmetry	P1	P1	P1		P1	P1	P1
Models generated	13	13	13		13	13	13
Models included*^a^*	12	13	13		12	13	12
NSD_mean_	0.67 ± 0.04	0.49 ± 0.01	0.60 ± 0.02		0.56 ± 0.03	0.79 ± 0.04	0.77 ± 0.07
χ^2^ *versus* raw data	2.72	1.04	2.08		1.71	1.37	1.50

*^a^* Models discarded when NSD > NSD_mean_ + 2 ×S.D., *n* = 12–13.

We attempted to model the three component proteins and tRNA substrate into the complex envelopes. To do so, we first derived molecular envelopes for the individual proteins based on their own scattering data (Fig. S6, *A*, *B*, *E*, and *F*). The isolated MRPP1_ΔMTS_ was found to exist as both monomer and dimer in solution. The obtained SAXS envelope could therefore account for a dimer containing two MTase domains at opposite ends sandwiching two compact, interacting N-terminal domains in the center (Fig. 6*A*) or could alternatively comprise only one MTase domain at one end of the pocket with an elongated, flexible N-terminal domain taking up the remaining space. A similarly elongated SAXS envelope was seen for the monomeric Trm10 homologue of MRPP1 from yeast in solution (15). For MRPP2, a calculated scattering curve for the tetrameric crystal structure provides an excellent fit (χ^2^ = 0.952 for *q* values between 0.01 and 0.45°) to the scattering data (Fig. S8*B*), and the structure can be easily modeled into the low-resolution envelope (Fig. 6*B*). The modeled envelope for MRPP3_ΔMTS_ accommodates the reported crystal structure (aa 207–583, MRPP3_Δ206_) while leaving room only for the N-terminal extension (aa 51–206) not present in the structure (Fig. 6*C*). As a control, we measured scattering data for MRPP3_Δ206_ protein, which shows a good fit (χ^2^ = 1.74 for *q* values between 0.01 and 0.45°) to the structure (Fig. S8*C*). Both MRPP3_ΔMTS_ and MRPP3_Δ206_ envelopes indicate a monomeric protein that is supported by analytical SEC (Fig. S4*F*) and in line with MRPP3 orthologues from *A. thaliana* (PRORP1 and PRORP2) (42).

With the above models in place, we docked the component subunits incrementally into the complex envelopes. The MRPP1_ΔMTS_–MRPP2 envelope was processed in both P1 and P2 point groups to account for monomeric and dimeric MRPP1_ΔMTS_ protein, respectively. The molecular mass derived from the scattering curve of 190 kDa is consistent with the SEC-MALS data, agreeing with a 2 MRPP1 to 4 MRPP2 stoichiometry. The envelope obtained from processing in P2 contains a globular region in the center, where one MRPP2 tetramer can be fitted, and two smaller peripheral regions sandwiching MRPP2, where each region could accommodate one MRPP1 monomer (Fig. 6*E*). The envelope from processing in P1 shows a similar globular region corresponding to the MRPP2 tetramer, but also an extended region representing an MRPP1 dimer (Fig. 6*D*). When comparing the Kratky plots for isolated MRPP1_ΔMTS_ and MRPP1_ΔMTS_ complexed with MRPP2, it can be easily seen that any flexibility found in isolated MRPP1_ΔMTS_ is lost when bound to MRPP2, giving rise to a more compact structure (Fig. S8*D*).

Adding pre-(mt)tRNA^Ile^ to the binary complex, processed without enforced symmetry, reveals a lobe consistent with the size of one pre-(mt)tRNA^Ile^ molecule (Fig. 6*F*). This lobe is in proximity to both MRPP1_ΔMTS_ and MRPP2 regions, which have altered their relative orientations in the MRPP1_ΔMTS_–MRPP2–tRNA complex, compared with the MRPP1–MRPP2 complex. The pre-(mt)tRNA^Ile^ cannot be modeled accurately due to the large 5′- and 3′-overhangs, but the presumed position can be in close proximity to the m^1^R9 MTase active site and an N domain, consistent with our finding that the N domain of MRPP1 alone can bind tRNA.

The MRPP1_ΔMTS_–MRPP2–MRPP3_ΔMTS_–tRNA complex was prepared by reconstituting MRPP1_ΔMTS_–MRPP2 with pre-(mt)tRNA^Ile^ in the presence of Mg^2+^ and a catalytic mutant MRPP3(D479A) (43, 44) (Fig. S4*D*) used to prevent turnover of the pre-(mt)tRNA^Ile^, which may dissociate the complex. The *ab initio* model generated from the scattering data with no enforced symmetry reveals a lobe adjacent to the putative MRPP2 and tRNA regions, which can accommodate one MRPP3_ΔMTS_ molecule (Fig. 6*G*). Thus, our model proposes a stoichiometry of 2 MRPP1_ΔMTS_, 4 MRPP2, 1 MRPP3_ΔMTS_, and 1 pre-(mt)tRNA^Ile^ molecule. This model would suggest a direct interaction between MRPP2 and MRPP3_ΔMTS_, possibly without MRPP1_ΔMTS_ involvement. Nevertheless, size-exclusion chromatography of a mixture containing MRPP2 and MRPP3_ΔMTS_ (in the absence of pre-(mt)tRNA^Ile^ substrate) did not reveal the presence of a stable subcomplex (Fig. S4*E*).

## Discussion

In this study, we characterized biochemically and structurally the MRPP1 protein and its interaction with MRPP2, MRPP3, and tRNA. This work reveals the first structural models for the unique binary and ternary complexes that are responsible for mitochondrial tRNA processing.

The eukaryote-only MRPP1 appears to exist as both monomer and dimer in solution, with dimerization taking place via the N domain. The prompting of dimerization by the N-terminal domain contrasts with canonical SPOUT from other subfamilies (*e.g.* TrmL and TrmH), where homodimerization takes place via their MT domains. Our structure explained why the conventional SPOUT dimer interface is absent in MRPP1. The dimerization of eukaryote-only MRPP1 also contrasts with other characterized Trm10 members from Archaea and lower eukaryotes that clearly exist as monomers in their full-length proteins (15, 35). Like MRPP1, the MT-domain topology of these members also prohibits SPOUT-like dimerization. The known Trm10 family members also contain N and C extensions of different lengths, in addition to their MT domain, displaying low sequence identity, which might explain the unique self-interaction found for this domain in MRPP1.

To date, the Trm10 members have been characterized to be m^1^G9-specific (*S. cerevisiae* and *S. pombe*), m^1^A9-specific (*S. acidocaldarius*), and m^1^A9/m^1^G9 dual-specific (MRPP1 and *T. kodakaraensis*) MTases. The MRPP1–MRPP2 complex harboring either D314N or Q226A substitution on MRPP1 demonstrated no m^1^A9 and m^1^G9 MTase activity, thus confirming essential catalytic roles for these strictly invariant residues. During methyl transfer, Asp-314 can activate the N1 atom of G9 and exocyclic N6 atom of A9 to launch a nucleophilic attack on the reactive methyl group of SAM. The catalytic role of Gln-226 is less clear. Ala substitution of the equivalent Gln residue in yeast Trm10 showed that pre-(mt)tRNA or SAM binding was unaffected (15). Instead, Gln-226 could be involved in binding the phosphate group in purine-9 of the (mt)tRNA, as suggested for a corresponding Asn residue in TrmL and TrmH from bacteria (45, 46). The molecular basis of purine specificity in the Trm10 family will be clarified by substrate-bound crystal structures in the future.

The fact that MRPP1 requires an additional partner protein for catalysis contrasts with all functionally characterized Trm10. For example, human cytosolic TRMT10A alone can catalyze m^1^G9 formation *in vitro* (16). The functional discrepancy is probably due to differentiating features in the N- and C-terminal extensions of the MT domains, as this domain is highly superimposable among Trm10s. Our SPOT analysis identifies regions from both the N and MT domains of MRPP1 to be involved in MRPP2 interaction. These sequentially disjoined regions can spatially orient into one pan-interaction patch or interact with MRPP2 as separate modules. Two missense mutations in MRPP1 (R118L and T272A), reported to cause a rare systemic mitochondrial disease (19–21), are located within this putative interaction patch, suggesting that these mutations could affect complex formation. We detected a direct interaction between the N domain of MRPP1 and tRNA, and a lack of this domain also abolished dimerization and MTase activity.

In addition to assistance in catalysis, the interaction with MRPP2 also affects the structural integrity of MRPP1, as suggested before (40). Our co-expression experiment suggests that the stability of MRPP1, particularly its N domain, toward proteolytic degradation depends on the presence of MRPP2. A comparison of SAXS data (Kratky plot) between MRPP1 alone and MRPP1 complexed with MRRP2 showed that MRPP1 becomes structurally more compact when bound to MRPP2, whereas MRPP1 is prone to degradation in the absence of MRPP2. We therefore speculate that the dimer formation of MRPP1 alone via the N domain is an *in vitro* observation induced by the absence of MRPP2. Such self-interaction of MRPP1 in a dimer would stabilize an otherwise monomeric MRPP1 protein, preventing its degradation at the N domain. We would further envisage that in the cell, MRPP1 would largely be present in complex with MRPP2.

Our combinatorial solution studies (SAXS and SEC-MALS) revealed a 2:4 stoichiometry for the MRPP1–MRPP2 complex, as suggested previously (28, 47). We envisage two possible subunit arrangements, backed by SAXS data processing, that could account for such stoichiometry. On the one hand, the SAXS envelope processed with *P2* symmetry favored the fitting of two MRPP1 monomers on distal sides of the MRPP2 tetramer in a symmetrical manner. On the other hand, the SAXS envelope without enforced symmetry would suggest binding of an MRPP1 homodimer to only one face of the MRPP2 homotetramer, implying that MRPP1 would contact MRPP2 in an asymmetric manner, a scenario that is difficult to reconcile, considering the symmetrical shape of MRPP2 homotetramer. Future high-resolution structural studies to resolve the two possibilities are clearly warranted.

SAXS studies on MRPP1–MRPP2–tRNA complex, processed without forced symmetry, suggested one pre-(mt)tRNA molecule bound per MRPP1–MRPP2 complex and that tRNA binding could rearrange the relative orientation between MRPP1 and MRPP2. The stoichiometry of one tRNA substrate per MTase dimer has been shown for TrmH and TrmD (45, 48, 49). EMSA showed that MRPP1, similarly to TrmD and TrmH, involves regions beyond the MT domain for binding pre-(mt)tRNA, although the N-terminal non-MT regions in TrmD and TrmH are significantly shorter than the MRPP1 N domain. The assistance in tRNA binding by regions beyond the MT domain is shown for many SPOUT members (15, 35, 48, 50). It is possible that these non-MT domains are tasked to recognize more universally conserved parts of the tRNAs if the immediate environment around the modified nucleotide, in contact with the MT domain, shows low structural conservation.

A direct interaction between MRPP2 and the (mt)tRNA substrate has previously been suggested, based on the discovery that NAD^+^/NADH-binding Rossmann folds could accommodate RNA (51, 52) and on *in vivo* interaction data (53). We, in addition to Vilardo *et al.* (16), however, did not detect any direct interaction *in vitro* between recombinant MRPP2 and pre-(mt)tRNA using EMSA. It is currently unclear how the MRPP1–MRPP2 interaction would influence the binding of the (mt)tRNA substrate. A small increase in the dissociation constant (*K_D_*) of MRPP1 toward the mature (mt)tRNA with the addition of MRPP2 was observed previously using an EMSA (16). Therefore, MRPP2 may account for the *K_D_* change by 1) assisting in direct interaction with (mt)tRNA, 2) stabilizing a catalytically important structural element in MRPP1 (*e.g.* the N domain shown to be involved in pre-(mt)tRNA binding), or 3) “remolding” the catalytic components of the MRPP1 MT domain. Future studies are required to further explore these possible mechanisms.

The MRPP1–MRPP2–MRPP3 complex is formed only in the presence of the pre-(mt)tRNA substrate. This reconstituted ternary complex, but not MRPP3 alone, exhibited RNase P activity toward the tested pre-(mt)tRNA^Ile^ substrate. No RNase P activity was observed for the ternary complex in the absence of either the PPR-domain or the core/NYN domains of MRPP3, but partial removal of the N extension (43 residues) still left a functional protein. Again, it is intriguing that the RNase P activity of MRPP3 depends so strongly on additional protein partners, when MRPP3 homologues from lower eukaryotes efficiently perform the 5′-end processing on their own (54, 55). The SAXS model obtained without enforced symmetry supported a stoichiometry of MRPP1 (dimer):MRPP2 (tetramer):MRPP3 (monomer):pre-(mt)tRNA (one molecule). MRPP3 alone is a monomer in solution and in the crystal, and it is therefore not surprising that it could exist as such in the complex.

(mt)tRNAs often adopt “bizarre” structures, especially before they undergo posttranscriptional modifications. It is possible that the MRPP1–MRPP2 complex activates the pre-(mt)tRNA for recognition by MRPP3, by remolding the (mt)tRNA in a way that offers a similar recognition surface for MRPP3 across all bizarre (mt)tRNAs. The role of MRPP1–MRPP2 as the pre-(mt)tRNA recognition component of the RNase P complex is in line with findings herein that the MRPP1–MRPP2 complex can bind the pre-(mt)tRNA before binding of MRPP3. Structural studies of MRPP3 alone have revealed the absence of Mg^2+^ cofactor in the active site (43, 44). Binding of MRPP1–MRPP2 and/or tRNA could therefore configure MRPP3 into a conformation that allows binding of the Mg^2+^ cofactor necessary for pre-(mt)tRNA 5′-end processing.

## Experimental procedures

### Cloning, expression, and purification of MRPP1, MRPP2, and MRPP3

DNA fragments encoding human MRPP1, MRPP2, or MRPP3 (MGC collection 4719195, 2819721, or 5206545) were subcloned into pNIC28-Bsa4 vector (GenBank^TM^ EF198106) incorporating an N-terminal His_6_ tag. Constructs were generated of MRPP1 covering residues 40–403 (MRPP1_ΔMTS_), 40–203 (MRPP1_N_), 195–403 (MRPP1_MT+C(Δ194)_), 203–403 (MRPP1_MT+C(Δ202)_) and of MRPP3 covering residues 51–583 (MRPP3_ΔMTS_), 95–583 (MRPP3_Δ94_), 86–274 (MRPP3_N_), and 274–583 (MRPP3_C_). To generate an MRPP1–MRPP2 co-expression plasmid, a DNA fragment of human MRPP1 and MRPP2, interspersed with a ribosome-binding site, was subcloned into the pNIC28-Bsa vector. MRPP1 and MRPP3 mutations were generated using the QuikChange site-directed mutagenesis kit (Agilent), based on the pNIC28-Bsa4 vector containing full-length His_6_-MRPP3 alone (for MRPP3) or both mature His_6_-MRPP1 and full-length MRPP2 in a bicistronic transcript (for MRPP1). The presence of the DNA changes was verified by Sanger sequencing.

Plasmids were transformed into *E. coli* BL21(DE3)-R3-pRARE2 competent cells, cultured in Terrific Broth at 37 °C, and protein expression was induced with 0.1 mm isopropyl β-d-1-thiogalactopyranoside for overnight growth at 18 °C. For small-scale expression and solubility test, cell pellets from 50-ml cultures were lysed by sonication and clarified by centrifugation, and the clarified lysate was applied to an immobilized metal affinity column (IMAC) for protein purification.

For large-scale purification for biochemical assays, MRPP2 (WT and mutants), co-expressed MRPP1–MRPP2 complex, and MRPP3 cell pellets from 3–12-liter cultures were lysed as described above, and recombinant proteins were purified by IMAC and size-exclusion chromatography, treated with tobacco etch virus protease overnight, and further purified by reverse IMAC and ion-exchange chromatography eluted with a NaCl gradient (supporting Experimental procedures for protein purification).

### In vitro transcription of mitochondrial tRNA precursors

cDNAs representing human mitochondrial tRNA^Ile^ and tRNA^Val^ precursors were designed with leader and trailer sequences of 30 and 15 nucleotides, respectively, and *in vitro* transcribed from a PCR-amplified template by T7 RNA polymerase using the Transcript Aid T7 high-yield transcription kit (Thermo Scientific). The transcribed precursor tRNAs were purified using the RNeasy Mini Kit (Qiagen).

### Crystallization, data collection, and structure determination

Crystals of hMRPP1_MT+C(Δ202)_ were grown by vapor diffusion at 4 °C, from sitting drops composed of 200 nl of protein (11 mg/ml; preincubated with 4.8 μm SAM) and 100 nl of reservoir solution containing either 14% PEG 1000, 28% glycerol or 12% PEG 1000, 28% glycerol, 1.5% (w/v) PEG 3350. Crystals were cryo-protected with reservoir solution supplemented with 25% (v/v) propylene glycol and flash-cooled in liquid nitrogen. Heavy atom (HA)-derivatized crystals were obtained by soaking with 10 mm C_9_H_9_HgNaO_2_S for 1 h, 10 min. Diffraction data were collected on the Diamond Light Source beamline for the HA-soaked crystals to 2.49 Å and for two crystals of the as-purified protein to 2.22 and 1.96 Å. Phases were determined by SIRAS with the SHELX package (56) using the HA data set. This procedure generated an initial polyalanine model covering the entire protein chain. The phases from the HA data set were combined with the 2.22 Å native data set, and the model was completed with side chains assigned to sequence in COOT (57). This model was used as search model in a molecular replacement done in PHASER (58) in the CCP4 suite of programs (59) using the native data collected to 1.96 Å. Modeling and refinement were carried out using Refmac (60) and Coot (61). There was no electron density for residues 380–403 and lower than average electron density for residues 343–348 in all chains.

### Analytical SEC

100-μl protein samples of varying concentrations were injected onto a 24-ml Superdex S200 10/300 GL column (GE Healthcare) or a KW403-4F column (Shodex). Fractions were collected, and TCA was precipitated before analysis on a 4–12% bis-tris polyacrylamide gel (Invitrogen). Molecular weight standards from Bio-Rad, containing thyroglobulin (670 kDa), γ-globulin (158 kDa), ovalbumin (44 kDa), and myoglobin (17 kDa), and vitamin B_12_ (13.5 kDa) were used for calibration (Fig. S1*A*). Complex formation and molecular weights were determined from experimental replicates of *n* = 3.

### DSF

DSF was performed in a 96-well plate using an Mx3005p RT-PCR machine (Stratagene) with excitation and emission filters of 492 and 610 nm, respectively. Each well consisted of 2 μl of protein in a buffer of 150 mm NaCl, 10 mm HEPES, pH 7.5, to a final concentration of 2 μm, 2 μl SYPRO ORANGE diluted 1000-fold in buffer from the manufacturer's stock (Invitrogen), and (if applicable) 2 μl of ligand at various concentrations. Fluorescence intensities were measured from 25 to 96 °C with a ramp rate of 3 °C/min. *T_m_* was determined by curve fitting using GraphPad Prism version 5.01 software (62). Data were evaluated using a standard P-test according to a standard procedure performed using the GraphPad Prism version 5.01 software.

### SPOT blot assay

15-Mer peptides of mature MRPP1 (residues 40–403), with maximum overlap of 13 amino acids from the peptide before or after (*i.e.* a two-amino acid shift in register), were synthesized on cellulose membranes using a MultiPep SPOT peptide arrayer (Intavis). In the synthesis, some spots were left blank (no peptide coupled), and some were coupled only to His_6_ to serve as controls. His_6_-tagged MRPP2 was added to a final concentration of 1 μm and detected with horseradish peroxidase–conjugated anti-His antibody in a 1:3000 dilution in 2.5% BSA PBST buffer. Blots were developed using an ECL kit (Thermo Scientific) with equal amounts of peroxide solution and luminol enhancer solution. Membrane spots were visualized with an ImageQuant Las-4000 camera (Fujifilm) on the chemiluminescence setting. Densitometry was performed using E-editor version 2.0 software.

### tRNA binding by EMSA

EMSA was performed in a 12-μl reaction mixture: 100 mm NaCl, 50 mm Tris-HCl, pH 8.0, 3 mm DTT, 3 mm MgCl_2_, 1 μm tRNA, and the proteins at 9–16 μm. Reaction mixtures were incubated on ice for 10 min, mixed with 6 μl of EMSA loading buffer, and applied to a nondenaturing 3–8% Tris acetate polyacrylamide gel (NuPAGE, Invitrogen) along with a sample of protein markers (NativeMark^TM^ unstained protein ladder, Thermo Fisher Scientific) and electrophoresed in Tris/glycine buffer at 4 °C for 2 h, 40 min at a constant voltage of 150 V. Subsequently, gel was stained with SYBR Gold (1:1000 dilution in Tris/glycine buffer) for 30 min and imaged using the Bio-Rad ChemiDoc imaging system. Coomassie Brilliant Blue (Generon) was used to subsequently stain gel for protein overnight and was visualized using the Bio-Rad ChemiDoc imaging system.

### Methyltransferase activity assay

1 μCi of [*methyl*-^3^H]SAM (15 Ci/mmol) (PerkinElmer Life Sciences), 1 μm individual or complexed protein(s), and 1 μg of *in vitro* transcribed pre-(mt)tRNA was diluted in a buffer of 50 mm Tris-HCl, pH 8, 5 mm MgCl_2_, 20 mm NaCl, and 1 mm DTT to 200 μl. The solution was incubated for 60 min at 37 °C in a heat block. The reaction was stopped by phenol extraction, and the pre-(mt)tRNA substrate was TCA-precipitated. Radioactive methylated pre-(mt)tRNA was captured on a Whatman glass microfiber GF/C filter (GE Healthcare) and washed three times with ethanol before the measurement of radioactivity in a scintillation counter.

### RNase P activity assay

RNase P cleavage was performed by mixing 300 nm MRPP1–MRPP2, 150 nm MRPP3, 10 units of RNase inhibitors (RNasin from Promega), and 400 nm *in vitro* transcribed pre-(mt) tRNA^Ile^ in a buffer of 30 mm Tris-HCl, pH 8, 40 mm NaCl, 4.5 mm MgCl_2_, and 2 mm DTT to a total reaction volume of 8.25 μl. The reaction was performed at room temperature and stopped at set times by transferring 1 μl of the reaction mixture into 5 μl of 500 mm EDTA and heating to 95 °C. This sample was analyzed by denaturing PAGE on a 6% TBE-urea gel (Invitrogen) run in TBE buffer (Invitrogen). The gel was stained for 30 min with SYBR Gold (Invitrogen) diluted 1000-fold in TBE buffer from the manufacturer's stock and imaged using the Bio-Rad ChemiDoc imaging system.

### SEC-SAXS

Proteins were subjected to SEC-SAXS on a KW403–4F column (Shodex) at 4.5 mg/ml in 50 mm sodium phosphate, pH 7.4, 300 mm NaCl, 5% glycerol, 1% sucrose, 0.5 mm TCEP (for MRPP1, MRPP2, and MRPP1–MRPP2) or 100 mm NaCl, 50 mm Tris-HCl, pH 8, 3 mm MgCl_2_, 3 mm DTT (for MRPP1–MRPP2–pre-(mt)tRNA^Ile^ with and without MRPP3), by flowing sample through an in-vacuum quartz capillary of 1.6-mm diameter at 0.16 ml/min. Data were collected using a Pilatus2M detector (Dectris, Baden-Daettwil, Switzerland) with a sample–detector distance of 3914 mm and a wavelength of λ = 1 Å. The range of momentum transfer of 0.0041 < *s* < 0.41 Å^−1^ was covered (*s* = 4πsinθ/λ, where θ is the scattering angle). The data covering the peak were radially averaged, and the scattering of buffer was subtracted using Scatter (version 3.0) (Robert Rambo, http://www.bioisis.net)^6^ (65). The forward scattering *I*(0), Guinier *R_g_*, real-space *R_g_*, and Porod volume were obtained using Scatter (version 3.0), in which the *P*(*r*) distribution function and the *D*_max_ were also analyzed, before *ab initio* modeling was performed with the ATSAS suite of programs.

The identified stoichiometry of 2 MRPP1 to 4 MRPP2 for the binary complex from SEC-MALS offers the potential point symmetry P1, P2, or P4. In the *ab initio* modeling, 13 initial models were built using DAMMIF in fast computation mode with all three point groups. The NSD values were calculated for each generated model using SUPCOMB and subsequently compared to find the most probable ones using DAMSEL, with exclusion of models with NSD > mean + 2 × S.D. The most probable models were aligned using DAMSUP, averaged using DAMAVER, and filtered according to volume by DAMFILT (63). From the filtered model, a core structure was generated in DAMSTART from which a refined model was built in slow computational mode using DAMMIN, with *P1*, *P2*, or *P4* point symmetry (64). The scattering data for the MRPP1–MRPP2 complex processed in all three point groups (Table S1 and Fig. S8*A*) were compared with *ab initio* models for the separate protein components to identify the correct model as P1 or P2 (Fig. 6, *D* and *E*). Protein complexes including pre-(mt)tRNA were subsequently processed in *P1* only, as no specific symmetry could be assumed in such a multispecies arrangement (Fig. 6, *F* and *G*). Protein *M*_r_ values were determined using the shape- and fold-independent volume of correlation (*V_c_*)-based approach in Scatter (version 3.0) (65) (MRPP1_ΔMTS_–MRPP2) or determined directly from the scattering curve using SAXS MoW2, with the assumption that the proteins adopt a globular structure (MRPP2) (66).

### SEC-MALS

MALS studies were performed in-line with SEC on protein samples to assess monodispersity and mass of the SAXS samples using an 18-angle DAWN HELEOS light-scattering (LS) detector in which detector 12 was replaced with a DynaPro quasi-elastic light-scattering detector (Wyatt Technology). Simultaneous concentration measurements were made with an Optilab rEX refractive index detector (Wyatt Technology) connected in tandem to the LS detector. The MALS system was calibrated with BSA and glucose isomerase at 10 mg/ml to determine delay times, band broadening, and refractive index increment. Chromatographic separation was performed with a KW403–4F column (Shodex) at 4.5 mg/ml in 50 mm sodium phosphate, pH 7.4, 300 mm NaCl, 5% glycerol, 1% sucrose, 0.5 mm TCEP (for MRPP1, MRPP2, and MRPP1–MRPP2) using a flow rate of 0.250 ml/min.

## Author contributions

U. O. and W. W. Y. conceived the project. S. O. carried out the protein purification, crystallization, structure determination, and biochemical experiments. M. R. and L. D. contributed to the methyltransferase assay. F. F. contributed to protein purification and crystallization. J. K. and J. A. N. contributed to structure determination. S. O., R. P. R., H. J. B., W. G. N., A. A., J. Z., U. O., W. W. Y. analyzed data and contributed to the writing of the manuscript. All authors reviewed and approved the manuscript.

## Supplementary Material

Supporting Information

## Footnotes

6 Please note that the JBC is not responsible for the long-term archiving and maintenance of this site or any other third party hosted site.

## Footnotes

5 The abbreviations used are:

(mt)tRNA: mitochondrial tRNA
pre-(mt)tRNA: precursor (mt)tRNA
m^1^A9: 1-methyladenosine
m^1^G9: 1-methylguanosine
MTase: methyltransferase
SDR: short-chain dehydrogenase/reductase
MRPP: mitochondrial ribonuclease P protein
NYN: N4BP1 YacP-like nuclease
aa: amino acid(s)
MTS: mitochondrial targeting signal
SAM: *S*-adenosyl-l-methionine
DSF: differential scanning fluorimetry
SIRAS: single-isomorphous replacement with anomalous scattering
PDB: Protein Data Bank
SEC: size-exclusion chromatography
MALS: multiangle light scattering
SEC-MALS: SEC-coupled MALS
PPR: pentatricopeptide repeat
SAXS: small angle X-ray scattering
NSD: normalized spatial discrepancy
RMSD: root mean square deviation
EMSA: electrophoretic mobility shift assay
3α-HSD: 3α-hydroxysteroid dehydrogenase
HA: heavy atom
bis-tris: bis(2-hydroxyethyl)iminotris(hydroxymethyl)methane
TCEP: tris(2-carboxyethyl)phosphine
*M*_r_: molecular weight.
