# Supplementary material for: Structural insight into the human mitochondrial tRNA purine N1-methyltransferase and ribonuclease P complexes
Source: J Biol Chem. 2018 Jun 7;293(33):12862–76. doi: 10.1074/jbc.RA117.001286 (PMC6102140; doi:10.1074/jbc.RA117.001286)
Supplement: Supporting Information [file supp_293_33_12862__index.html]

Structural insight into the human mitochondrial tRNA purine N1-methyltransferase and Ribonuclease P complexes — Study of a methyltransferase and RNase P — Structural insight into the human mitochondrial tRNA purine N1-methyltransferase and ribonuclease P complexes — Study of a methyltransferase and RNase P — Supporting Information 

# Structural insight into the human mitochondrial tRNA purine N1-methyltransferase and ribonuclease P complexes

## Supporting Information

- Supporting information - Supporting information: Structural insight into the human mitochondrial tRNA purine N1-methyltransferase and Ribonuclease P complexes
