# Supplementary material for: Structural insight into the human mitochondrial tRNA purine N1-methyltransferase and ribonuclease P complexes
Source: J Biol Chem. 2018 Jun 7;293(33):12862–76. doi: 10.1074/jbc.RA117.001286 (PMC6102140; doi:10.1074/jbc.RA117.001286)
Supplement: Supporting Information [file supp_RA117.001286_134410_2_supp_148024_p9syds.docx]

Structural insight into the human mitochondrial tRNA purine N1-methyltransferase and Ribonuclease P complexes

**Stephanie Oerum^1^, Martine Roovers^2^, Robert P. Rambo^3^, Jola Kopec^1^, Henry J. Bailey^1^, Fiona Fitzpatrick^1^, Joseph A. Newman^1^, William G. Newman^4,5^, Albert Amberger^6^, Johannes Zschocke^6^, Louis Droogmans^7^, Udo Oppermann^1,8^, Wyatt W. Yue^1^**

^1^Structural Genomics Consortium, Nuffield Department of Clinical Medicine, University of Oxford, UK

^2^Institut de Recherches Microbiologiques Jean-Marie Wiame, Bruxelles, Belgium

^3^Diamond Light Source, Harwell Science and Innovation Center, Didcot, UK

^4^Division of Evolution and Genomic Sciences, Faculty of Biology, Medicine and Health, University of Manchester, Manchester, UK

^5^Manchester Centre for Genomic Medicine, Central Manchester University Hospitals NHS Foundation Trust, MAHSC, Manchester, UK

^6^Division of Human Genetics, Medical University of Innsbruck, Innsbruck 6020, Austria

^7^Laboratoire de Microbiologie, Universite libre de Bruxelles, Belgium

^8^Botnar Research Centre, NIHR Oxford Biomedical Research Unit, Oxford, UK

Running title: *Study of a methyltransferase and RNase P*

To whom correspondence should be addressed: Prof. Wyatt W. Yue, Telephone: +44 (0) 1865 617757; E-mail: wyatt.yue@sgc.ox.ac.uk. Prof. Udo Oppermann, Telephone: +44 (0) 1865 227308; E-mail: [udo.oppermann@sgc.ox.ac.uk](mailto:udo.oppermann@sgc.ox.ac.uk).

**Keywords:** Ribonuclease P, transfer RNA, RNA methyltransferase, RNA methylation, complex, TRMT10C, HSD10, MRPP, PRORP, HSD17B10.

**Supporting Information**

Experimental Procedures For Protein Purification

Supplementary Table S1

Supplementary Figure S1-S8

**EXPERIMENTAL PROCEDURES FOR PROTEIN PURIFICATION**

*Purification buffers*

Lysis buffer: 500 mM NaCl, 50 mM 4-(2-hydroxyethyl)-1-piperazineethanesulfonic acid (HEPES) pH 7.5, 5% glycerol, 0.5 mM [tris(2-chloroethyl) phosphate](https://en.wikipedia.org/wiki/Tris(2-chloroethyl)_phosphate) (TCEP), 20 mM imidazole

Wash buffer: 500 mM NaCl, 50 mM HEPES pH 7.5, 5% glycerol, 0.5 mM TCEP, 40 mM imidazole

Elution buffer: 500 mM NaCl, 50 mM HEPES pH 7.5, 5% glycerol, 0.5 mM TCEP, 250 mM imidazole

SEC buffer: 500 mM NaCl, 50 mM HEPES pH 7.5, 5% glycerol, 0.5 mM TCEP

Zero salt IEX buffer: 50 mM HEPES pH 6.8-7.5, 5% glycerol, 0.5 mM TCEP

Low salt IEX buffer: 50 or 300 mM NaCl, 50 mM HEPES pH 6.8-7.5, 5% glycerol, 0.5 mM TCEP

High salt IEX buffer: 2 M NaCl, 50 mM HEPES pH 6.8-7.5, 5% glycerol, 0.5 mM TCEP

Loading buffer: 1% w/v Sodium dodecyl sulphate (SDS), 31.5 mM Tris-HCl pH 6.8, 10% w/v glycerol, 180 mM dithiothreitol (DTT), 0.005% bromophenol blue

MES buffer: 0.1 M 2-(N-morpholino)ethanesulphonic acid (MES) pH 6.1

*Protein extraction for large-scale purification*

For MRPP1, MRPP2, MRPP3, and MRPP1-MRPP2 proteins, frozen cell pellets were thawed and resuspended on ice in 50 mL lysis buffer containing 1 mL protease inhibitor cocktail set III (Calbiochem) per L of harvested cell culture. The cells were lysed by passing five times on a homogeniser (EmulsiFlex-C5-Avestin) to a pressure of 1000 bar. The cell lysate was clarified by centrifugation (Beckman Coulter Avanti JXN-26) at 4ᵒC for 1 h at 16500 rpm. A sample of the centrifuged cell pellet was taken for SDS-PAGE and the supernatant was utilised for further purification.

*Immobilised metal affinity chromatography (IMAC)*

For MRPP1, MRPP2, MRPP3, and MRPP1-MRPP2 proteins, the clarified lysate from large-scale extraction was added with 1.25-3 mL of Ni-NTA affinity resin (GE Healthcare) and incubated at 4ᵒC for 1 h rotating at 8 rpm. The lysate, together with Ni-NTA resin, was centrifuged (Beckman Coulter Avanti JXN-26) for 30 min at 5000 rpm at 4ᵒC. The Ni-NTA pellet was applied onto a 1.5x10 cm (DxH) column (Bio-Rad) under gravity flow and the flow-through was collected for SDS-PAGE analysis. The Ni-NTA resin was washed with 2 x 10 CVs Lysis buffer and 2 x 10 CVs Wash buffer. The bound protein was eluted with 5-6 x 2 CVs of Elution buffer. 20 μL Lysis buffer was added with 0.75 μL of the captured flow-through and this sample, along with 20 μL of each wash and elution fraction, were subjected to SDS-PAGE. The fractions containing the protein of interest were pooled for further purification.

*Size exclusion chromatography (SEC)*

For MRPP1, MRPP2, MRPP3, and MRPP1-MRPP2 proteins, the pooled fractions from IMAC were concentrated to 5 mL using an Amicon Ultra centrifugal filter device (Merck Millipore) with a 10-30 kDa cut-off depending on the target protein size. The concentrated sample was loaded onto a 120 mL size exclusion chromatography (SEC) column (Sephadex 200 HiLoad 16/60 for which proteins, Sephadex 75 HiLoad 16/60 for which proteins or Superose 6 prep grade XK 16/70 for which proteins) connected to an AKTA-Express (GE Healthcare) in 4ᵒC. Before loading the protein, the column was pre-equilibrated with SEC buffer. Protein was loaded and eluted at a rate of 1 mL/min and 1.8 mL fractions were collected throughout the run. Protein was detected by UV absorbance and was observed in the chromatogram as an increase in absorbance (peak) when eluted. 20 μL of each peak fraction were subjected to SDS-PAGE. The fractions containing the protein of interest were pooled for further purification.

*Treatment with Tobacco etch virus protease*

For MRPP3 protein, the concentration of protein in the pooled fractions from SEC was determined. To remove the affinity tag from target proteins, Tobacco etch virus (TEV) protease was added to the eluates in a 1:20 (w/v) TEV-to-protein ratio and the solution was incubated overnight at 4ᵒC. 20 μL samples of before and after TEV protease treatment were evaluated for cleavage efficiency by SDS-PAGE.

*Reverse IMAC*

For MRPP3 protein, the TEV protease treated eluent was applied twice under gravity flow to a 2x10 cm column containing 125 μL Ni-NTA affinity resin (GE Healthcare) equilibrated with Lysis buffer and the flow-through was collected for SDS-PAGE. The Ni-NTA resin was washed with 2 x 8 CVs Lysis buffer and 2 x 8 CVs Wash buffer. TEV protease, and any uncut resin-bound protein, was eluted with 2 x 8 CVs Elution buffer. From SDS-PAGE analysis, fractions containing the protein of interest were pooled.

*Ion exchange chromatography (IEX)*

For MRPP1, MRPP2 and MRPP1-MRPP2 proteins, the pooled fractions from SEC were concentrated to 5 mL using an Amicon Ultra centrifugal filter device with a 10-30 kDa cut-off. The concentrated sample was diluted with Zero salt IEX buffer to 50 mL to reduce NaCl concentration and loaded onto an anion or cation chromatography column (HiTrap SP HP 5 mL/1 mL for which proteins, HiTrap Q HP 5 mL/1 mL for which proteins, Resource S 1 mL for which proteins or Resource Q 1 mL for which proteins) (GE Healthcare) connected to an AKTA-Purifier (GE Healthcare) at 4ᵒC. Before loading the protein, the column was pre-equilibrated with Low salt IEX buffer. The protein was loaded onto the column at 0.5 mL/min via a 50 mL superloop and the flow-through was collected for SDS-PAGE. The column-bound protein was eluted at a rate of 1 mL/min with a linear gradient of 0-35% High salt IEX buffer over 30-40 CVs. 1 mL fractions were collected throughout elution. 20 μL of the flow-through and peak fractions were subjected to SDS-PAGE.

|  | **MRPP1-MRPP2** | | | |
| --- | --- | --- | --- | --- |
|  | | | | |
| **Model building** | | | | |
| **Point symmetry** | | P1 | P2 | P4 |
| **Models gen.** | | 13 | 13 | 13 |
| **Models incl.*** | | 13 | 12 | 12 |
| **NSD_mean_** | | 0.56±0.03 | 0.93±0.29 | 1.19±+0.30 |
| **χ^2^ vs. raw data** | | 1.71 | 1.68 | 1.74 |

**TABLE S1** Statistics for ab initio model building in three different point symmetry groups (P1, P2 and P4) for data obtained from HPLC-SAXS of the complex between MRPP1ΔMTS and MRPP2. NSD: normalised spatial discrepancy. Models gen.: models generated. Models incl.: models included. *Models discarded when NSD > NSDmean + 2·SD, n = 12-13.


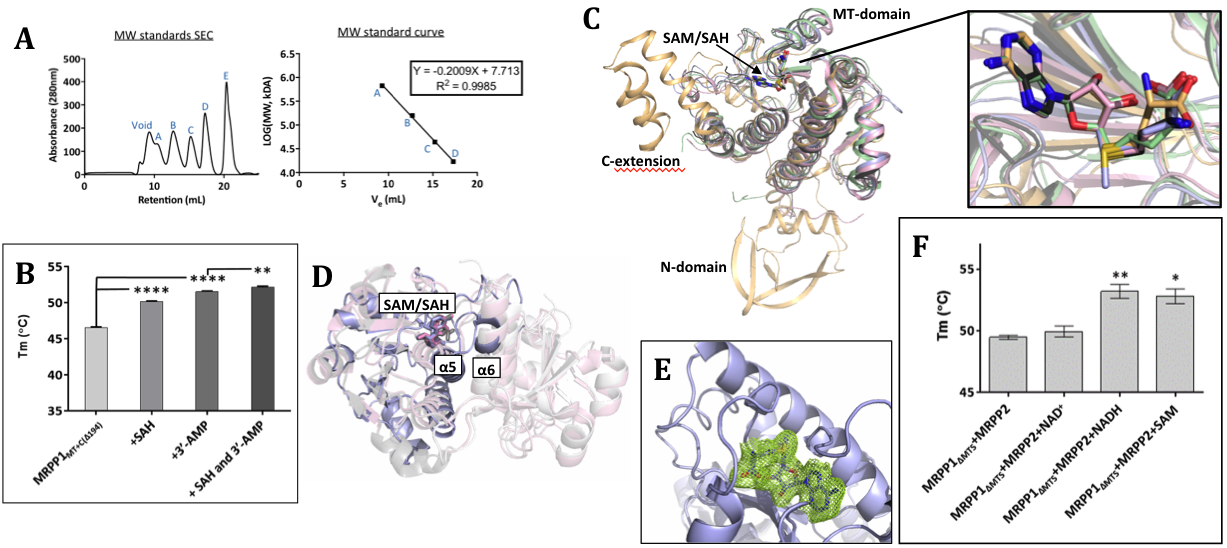


**FIGURE S1** *(A)* Left: Chromatogram of proteins with known MWs. A: thyroglobulin (bovine) MW=670 kDa, B: γ-globulin (bovine) MW=158 kDa, C: ovalbumin (chicken) MW=44 kDa, D: myoglobin (horse) MW=17 kDa, E: vitamin B_12_ MW=13.5 kDa. Right: LOG^10^MW plotted against V_e_ for each protein. Vitamin B_12_ was excluded from the plot due to the poor resolution of the S200 column in the lower MW-region. *(B)* Bar chart showing the melting temperature (Tm) from differential scanning fluorimetry of the MT-domain of MRPP1 (MRPP1_MT+C(Δ194)_) in the presence of S-adenosyl-homocysteine (SAH), adenosine-3’-monophosphate (3’-AMP) or both. ***P<0.01 and ****P<0.0001, n = 4*. *(C)* Structural alignment of MRPP1_MT_ (blue) and its closest structural homologues, including the MT-domains of human TRMT10A (PDB 4FMW) (black), Trm10 from *S. pombe* (4JWH) (green), Trm10 from *S. Cerevisiae* (4JWJ) (pink) and the full-length structure of Trm10 from *S. acidocaldarius* (5A7Y) (orange). Insert: alignment of the methylation donor S-adenosyl-L-methionine (SAM) from MRPP1_MT_ with the methylation product SAH from each homologue structure (shown in sticks, coloured as for the cartoon representation). *(D)* Helix α6 in the MT-domain of MRPP1_MT_ (blue) sterically blocks regular SPOUT family dimerisation otherwise occurring via helix α5 as shown for dimeric TrmL (PDB 4JAL) (grey) and TrmH (PDB 4KGN) (pink) from E. coli. *(E)* Difference electron density map (Fo-Fc, green, contoured at 2.0σ) for the SAM ligand in the crystal structure of MRPP1_MT_. *(F)* Melting temperature (Tm), investigated using differential scanning fluorimetry, of the complex between MRPP1_ΔMTS_ and MRPP2 in the presence and absence of ligands for SDR activity of MRPP2 (NAD^+^, NADH) or m^1^R9 MTase activity of the MRPP1_ΔMTS_ (SAM). **P<0.05 and **P<0.01*, n = 3.

*
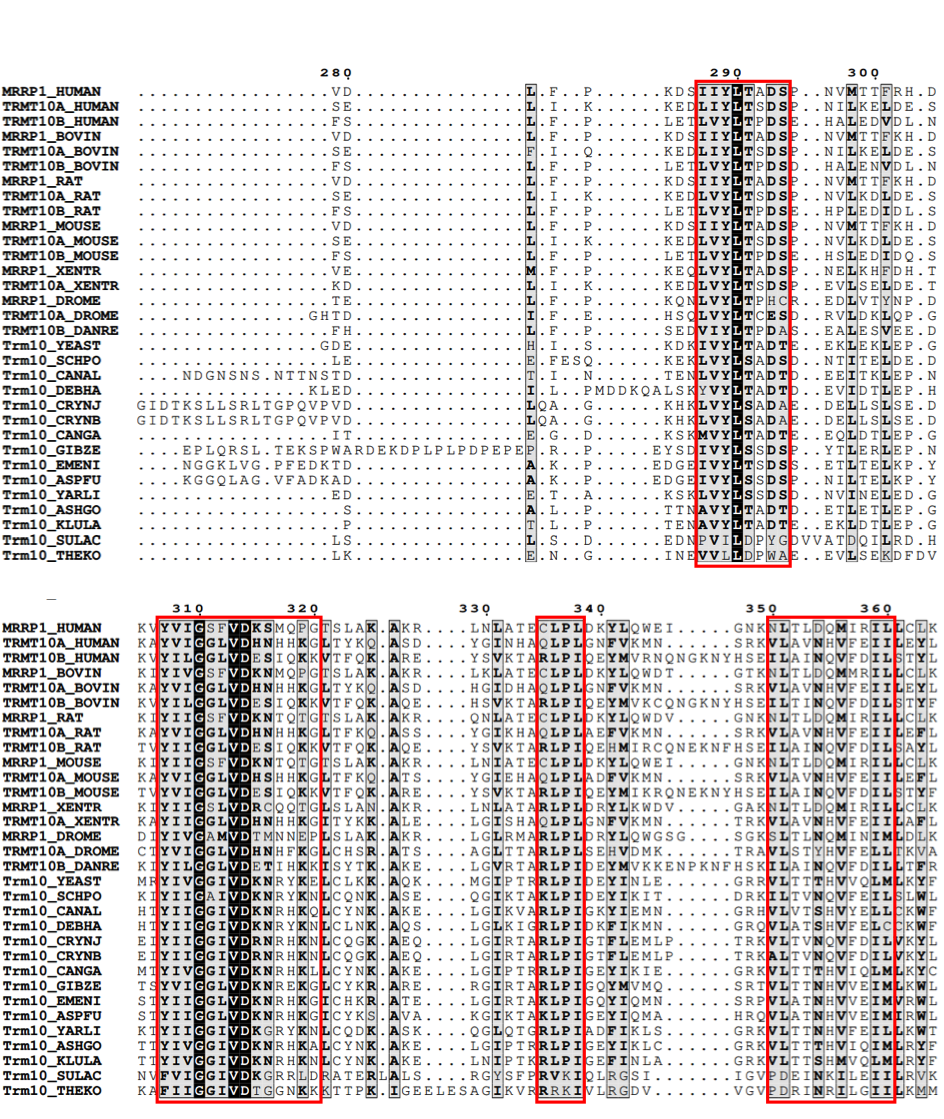
*

**FIGURE S2** Sequence alignment, generated with ESPript, of 32 Trm10 family members identified in UniProt (prosite entry: ps51675). For clarity, only the region corresponding to MRPP1_MT_ structure is shown. Identical and similar residues are boxed in black and grey, respectively. Red boxes indicate the four highly conserved sequences (motif I-IV) corresponding to loops enclosing the SAM ligand binding pocket. Residue numbering corresponds to human MRPP1. HUMAN: *Homo sapiens*, BOVIN: *Bos Taurus*, RAT: *Rattus norvegicus*, MOUSE: *Mus musculus*, XENTR: *Xenopus tropicalis*, DROME: *Drosophila melanogaster*, DANRE: *Danio rerio*, YEAST: *Saccharomyces cerevisiae*, SCHPO: *Schizosaccharomyces pombe*, CANAL: *Candida albicans*, DEBHA: *Debaryomyces hansenii*, CRYNJ: *Cryptococcus neoformans var. neoformans serotype D, strain JEC21 / ATCC MYA-565*, CRYNB: *Cryptococcus neoformans var. neoformans serotype D, strain B-3501A*, CANGA: *Candida glabrata*, GIBZE: *Gibberella zeae*, EMENI: *Emericella nidulans*, ASPFU: *Neosartorya fumigate*, YARLI: *Yarrowia lipolytica*, ASHGO: *Ashbya gossypii*, KLULA: *Kluyveromyces lactis*, SULAC: *Sulfolobus acidocaldarius*, THEKO: *Thermococcus kodakarensis*.


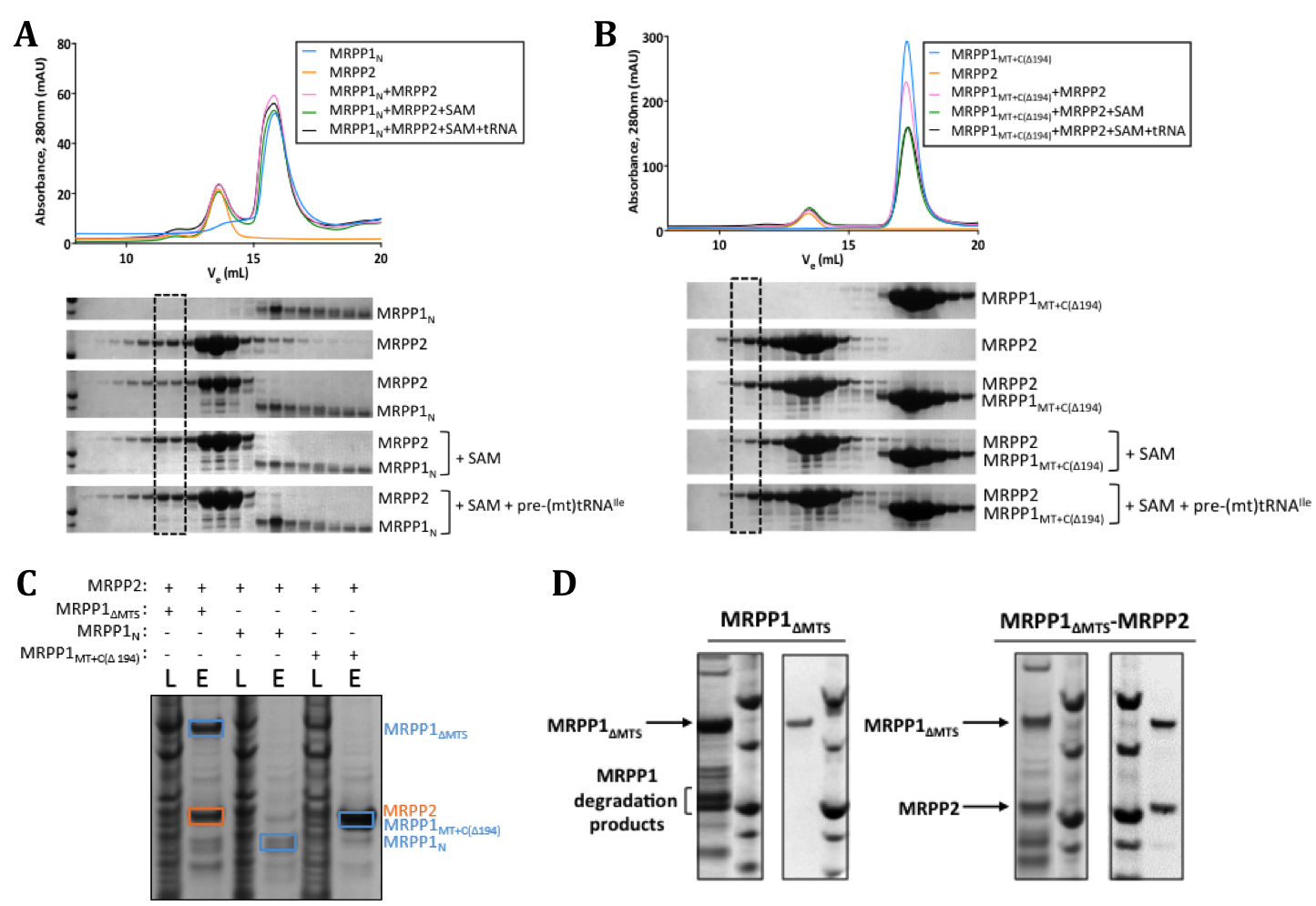


**FIGURE S3** *(A)* Size exclusion chromatography of MRPP1_N_ and MRPP2 applied separately (MRPP1_N_: blue line, MRPP2: orange line), and applied together (pink line) with SAM (green line) or with SAM and pre-(mt)tRNA^Ile^ (black line). The black box on SDS-PAGE indicates the elution fractions where MRPP1_N_ and MRPP2 would co-elute if a complex is formed. Gels visualised with a scanner of maximum 80 dpi resolution. *(B)* Size exclusion chromatography as for *A*, but using MRPP1_MT+C(Δ194)_ in place of MRPP1_N_. Gels visualised with a scanner of maximum 80 dpi resolution. *(C)* Nickel affinity pull-down of untagged MRPP2 co-expressed with His_6_-tagged MRPP1_ΔMTS_, MRPP1_N_, or MRPP1_MT_. The SDS-PAGE shows a diluted sample of the total lysate after sonication (L) and a sample of the protein elute from nickel affinity column (E). The orange box indicates the untagged MRPP2 protein bound to the column with His_6_-tagged MRPP1 proteins. The expressed MRPP1 proteins (MRPP1_ΔMTS_, MRPP1_N_, MRPP1_MT+C(Δ194)_) are indicated with blue boxes. *(D)* SDS-PAGE gels of the purified sample for MRPP1_ΔMTS_ expressed alone (left) and co-expressed with MRPP2 (right).


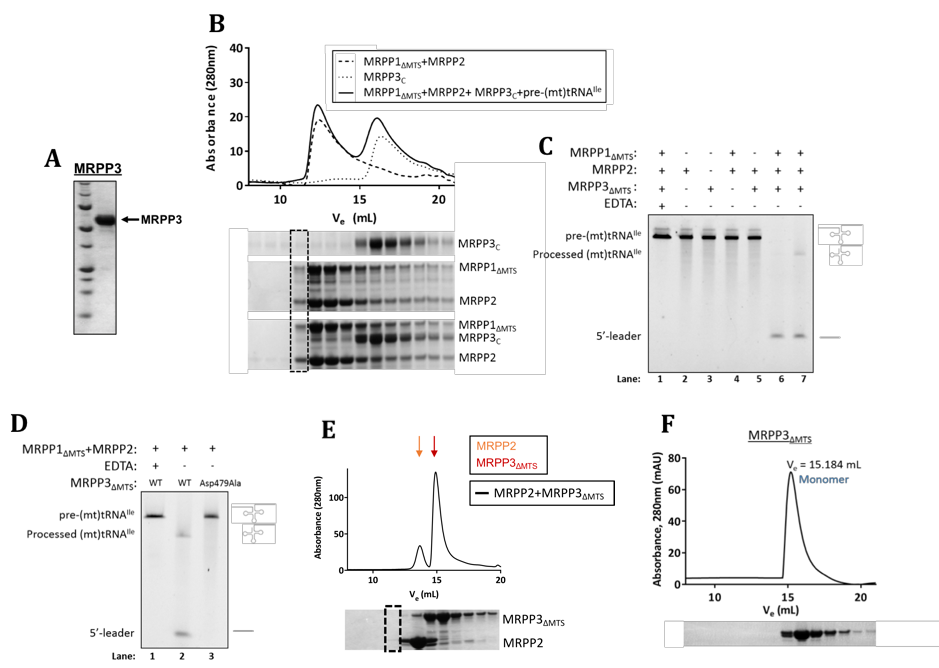


**FIGURE S4** *(A)* SDS-PAGE gels of the purified sample for MRPP3_ΔMTS_ (right) with standard protein markers (left). *(B)* Size exclusion chromatography on a Superdex S200 column for the complex of MRPP1_ΔMTS_ and MRPP2, the C-terminal region of MRPP3 (MRPP3_C_, residues 274-583) and a combination of these further added with pre-(mt)tRNA^Ile^. The SDS-PAGE shows lanes for each eluted fraction and the black box indicates where the proteins would co-elute if a ternary complex is formed with the addition of precursor tRNA. *(C)* UREA-PAGE denaturing gel, stained for RNA, with pre-(mt)tRNA^Ile^ and either MRPP2 alone (lanes 2), MRPP3_ΔMTS_ alone (lane 3), MRPP1_ΔMTS_ + MRPP3 (lane 4), MRPP2 + MRPP3_ΔMTS_ (lane 5), or MRPP1_ΔMTS_ + MRPP2 + MRPP3_ΔMTS_ (lane 6 and 7). Lane 1 is a negative control of MRPP1_ΔMTS_ + MRPP2 + MRPP3_ΔMTS_ added with EDTA before reaction start. Activity is indicated by downward movement of the precursor tRNA (top band) to a mature form of tRNA (mid-band) and the appearance of a small removed 5’-leader (bottom band). *(D)* UREA-PAGE denaturing gel assay as for *C*, but using the catalytic mutant Asp479Ala (lane 3) of MRPP3_ΔMTS_. A negative control with EDTA was included (lane 1) as in *C*. The intact ternary complex with wild-type (WT) MRPP3_ΔMTS_ was run as positive control (lane 2). *(E)* Size exclusion chromatography for the mixture of MRPP2 and MRPP3_ΔMTS_ proteins. The elution volumes for individual MRPP2 (from Fig. 3A) and MRPP3_ΔMTS_ (from Fig. S4F) proteins are indicated with arrows above the chromatogram (orange and red, respectively). The black box on SDS-PAGE of eluted fractions indicates the lane where both proteins should be present if a complex was formed. *(F)* Size exclusion chromatography of MRPP3_ΔMTS_. The elution volume (V_e_) and the corresponding oligomeric state, derived from a standard curve of proteins with known molecular weights, are indicated. The SDS-PAGE gel shows lanes of the eluted fractions.


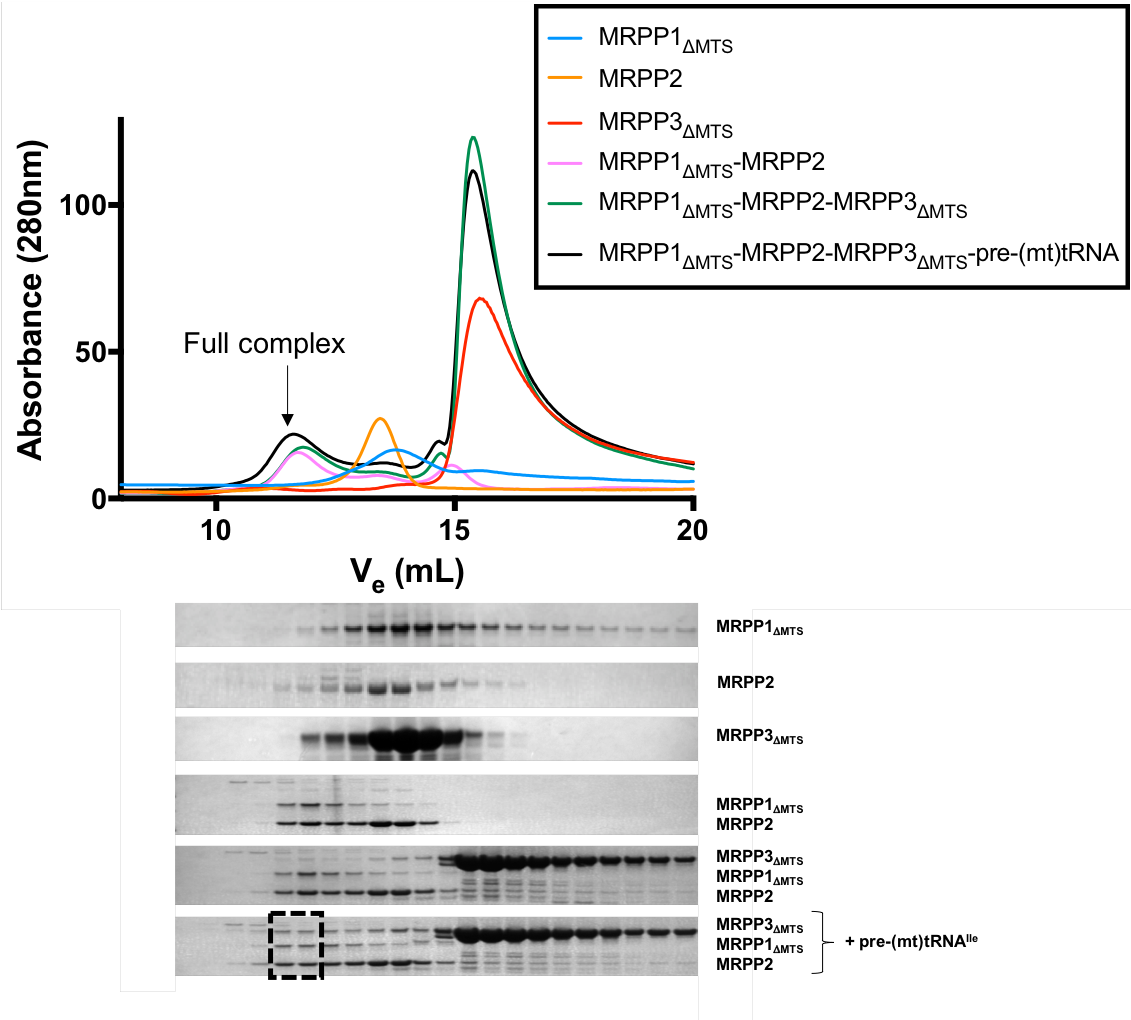


**FIGURE S5** Experimental replicate of Figures 3A and 5B. Analytical SEC profiles of individual MRPP1_ΔMTS_, MRPP2 and MRPP3_ΔMTS_ proteins (blue, orange and red line, respectively), mixture of MRPP1_ΔMTS_ and MRPP2 (pink line), mixture of MRPP1_ΔMTS_, MRPP2 and MRPP3 (green line), and mixture of MRPP1_ΔMTS_, MRPP2, MRPP3 and pre-(mt)tRNA^Ile^ (black line) applied to a Superdex S200 column. The black dashed box indicates lanes where the complex containing MRPP1_ΔMTS_, MRPP2, MRPP3_ΔMTS_ and pre-(mt)tRNA^Ile^ would be found on an SDS-PAGE gel of eluted fractions. Gels are SDS-PAGE with visualised proteins from each elution fraction along the chromatogram.

**
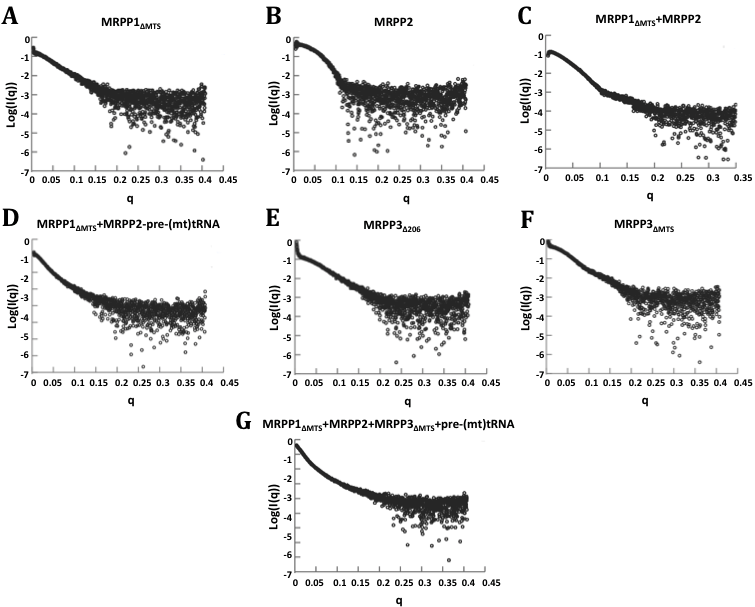
**

**FIGURE S6** Scattering curves (Log(q)/q) for *(A)* MRPP1_ΔMTS_, *(B)* MRPP2, *(C)* complex of MRPP1_ΔMTS_ and MRPP2, *(D)* complex of MRPP1_ΔMTS_, MRPP2 and pre-(mt)tRNA^Ile^, *(E)* MRPP3_Δ206_, *(F)* MRPP3_ΔMTS_, and *(G)* complex of MRPP1_ΔMTS_, MRPP2, pre-(mt)tRNA^Ile^ and MRPP3_ΔMTS_. All curves were obtained from HPLC-SAXS on a KW403-4F column at Diamond Lightsource beamline B21.

**
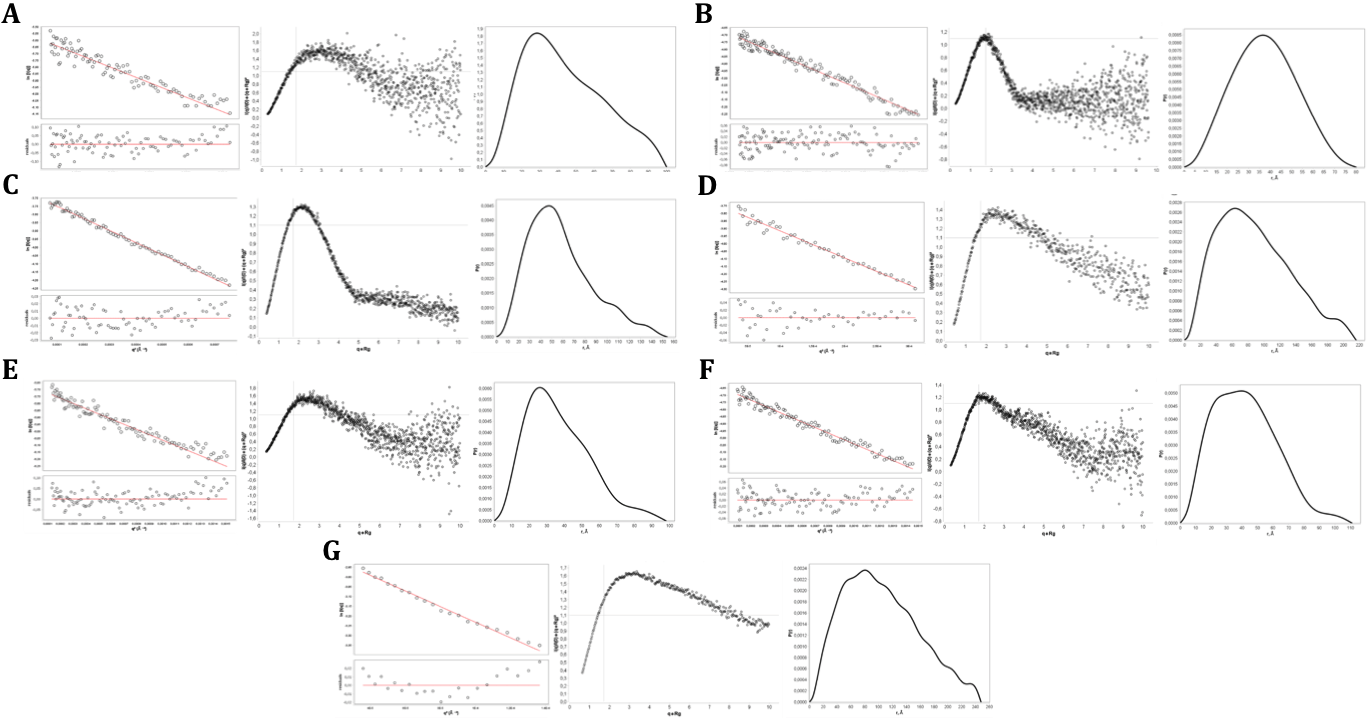
**

**FIGURE S7** Processing of scattering data obtained from HPLC-SAXS for MRPP1_ΔMTS_ *(A)*, MRPP2 *(B)*, complex of MRPP1_ΔMTS_ and MRPP2 *(C)*, complex of MRPP1_ΔMTS_, MRPP2 and pre-(mt)tRNA^Ile^ *(D)*, MRPP3_Δ206_ *(E)*, MRPP3_ΔMTS_ *(F)*, and complex of MRPP1_ΔMTS_, MRPP2, pre-(mt)tRNA^Ile^ and MRPP3_ΔMTS_ *(G)*. For each panel, the Guinier plot of ln(I(q)) vs. q^2^ (Å^-2^) (left, top), plot of residuals vs. q^2^ (Å^-2^) (left, bottom), Dimensionless Kratky plot of l(q)/I(0)*(q*R_g_)^2^ vs. q*Rg (middle), and P(r) distribution function of P(r) vs. r (Å) (right) are shown.


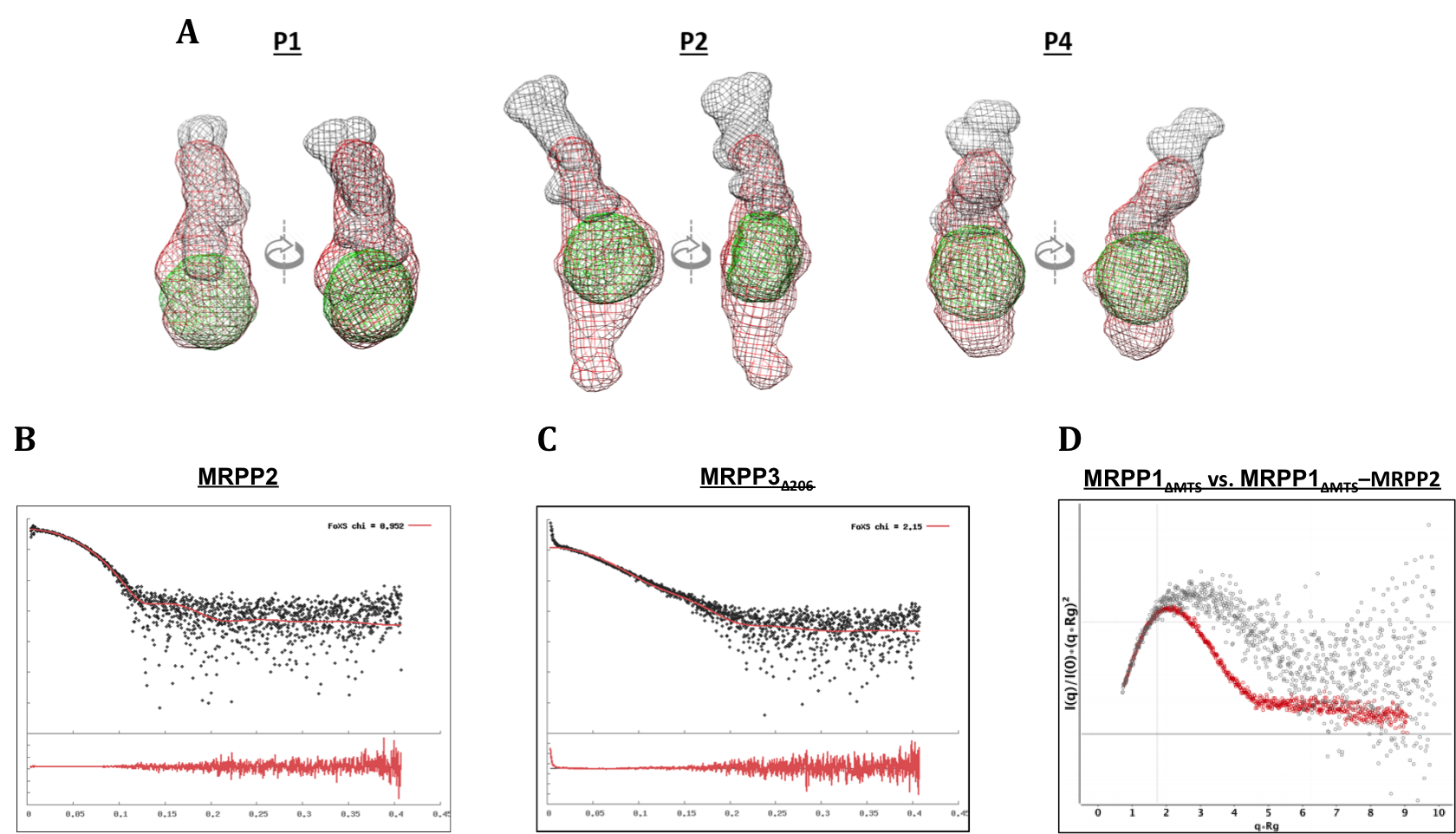


**FIGURE S8** *(A)* Envelopes generated from HPLC-SAXS scattering data of MRPP1_ΔMTS_ (grey mesh), MRPP2 (green mesh) and the complex of both (red mesh), calculated with *P1*, *P2* and *P4* point symmetry groups. The obtained envelopes for the individual proteins were computationally (MRPP2) or manually (MRPP1_ΔMTS_) fitted into the complex envelopes to provide the best fit. *(B)* Experimental scattering curve of MRPP2 (black) aligned with theoretical scattering curve (red) calculated from the crystal structure of MRPP2 (PDB: 1U7T). *(C)* Experimental scattering curve of MRPP3_Δ206_ aligned with the theoretical scattering curve (red) calculated from crystal structure of the same protein fragment (PDB: 4XGL). In the bottom of panels’ *B*-*C*, an indication of the fit between the theoretical and calculated scattering curves is shown, where large fluctuations indicate a poor fit. *(D)* Dimensionless Kratky plot for MRPP1_ΔMTS_ (grey) compared to MRPP1_ΔMTS_-MRPP2 (red).
